# Emerging robotic platforms in gynecologic surgery: a systematic review

**DOI:** 10.1007/s11701-026-03590-4

**Published:** 2026-06-22

**Authors:** Matteo Bruno, Davide Arrigo, Marco D’Indinosante, Emanuele Perrone, Nicolò Bizzarri, Giovanni Panico, Nicola Macellari, Matteo Figà, Mariano Giménez, Antonello Forgione, Jacques Marescaux, Anna Fagotti, Francesco Fanfani

**Affiliations:** 1 Dipartimento Scienze della Salute della Donna, del Bambino e di Sanità Pubblica, Fondazione Policlinico Universitario Agostino Gemelli, IRCCS, via Largo Francesco Vito 1, Rome, Italy; 2 Università Cattolica del Sacro Cuore, Roma, Italia; 3 Research Institute against Digestive Cancer, IRCAD, Strasbourg, France

**Keywords:** Robot-assisted surgery, Emerging robotic platforms, Gynecologic surgery, Minimally invasive surgery, Learning curve, Cost analysis

## Abstract

Robotic innovation in gynecologic surgery has expanded beyond established multiport ecosystems toward single-port, modular multi-arm, transvaginal, AI-enabled, and teleoperated platforms. However, available evidence remains fragmented and unsuitable for quantitative synthesis. This systematic review evaluated emerging robotic platforms in gynecologic surgery, focusing on procedure types, outcomes, costs, and learning curves. The review was prospectively registered in PROSPERO (CRD420261360389). Eligible studies included clinical studies evaluating emerging robotic platforms in gynecologic surgery. PubMed, Scopus, and Web of Science were searched on 4 April 2026. After duplicate removal, 408 records were screened, 160 full texts assessed, and 92 studies included. Data extraction and risk of bias assessment were performed independently in duplicate. The 92 studies covered 13 platforms and were mainly retrospective single-center studies. Evidence was most extensive for da Vinci SP and HUGO RAS, with more limited but informative data for Senhance, Versius, hinotori, and KANGDUO. Evidence for other systems consisted mainly of first-in-human, pilot, or feasibility studies. Hysterectomy and sacrocolpopexy were the most frequently reported procedures. Overall, perioperative outcomes and short-term safety appeared favorable, despite limited follow-up and heterogeneous outcome definitions. Cost data were mainly available for Senhance and KANGDUO. Learning-curve evidence was strongest for da Vinci SP and HUGO RAS. Risk-of-bias was predominantly moderate. Emerging robotic platforms have demonstrated broad technical feasibility and generally acceptable short-term perioperative safety in selected gynecologic procedures, but current evidence is heterogeneous, observational, and methodologically limited. Rigorous prospective studies, standardized economic analyses, longer follow-up, and learning-curve evaluations are needed before comparative conclusions can be drawn.

**Supplementary Information:**

The online version contains supplementary material available at 10.1007/s11701-026-03590-4.

## Introduction

 Robot-assisted gynecologic surgery is now an established minimally invasive option for a broad range of benign, reconstructive, and selected oncologic procedures. The principal advantages of robotic surgery are enhanced dexterity, tremor filtration, three-dimensional visualization, and improved surgeon ergonomics compared with conventional laparoscopy [1–3].

Over the past several years, the field has moved beyond the traditional multiport da Vinci ecosystem toward a heterogeneous generation of next-generation platforms that includes dedicated single-port systems, modular multi-arm open-console robots, transvaginal or natural-orifice devices, and systems designed for remote or hybrid operation [4–9].

These technologies should not be regarded as interchangeable devices. Their architecture, port strategy, docking logic, console configuration, and force-feedback characteristics differ substantially, and those differences may influence case selection, operating-room workflow, and overall procedural cost [5–8]. These distinctions are especially consequential in gynecology, where hysterectomy, myomectomy, adnexal procedures, sacrocolpopexy, endometriosis surgery, and oncologic staging each impose distinct technical demands on access geometry, triangulation, suturing mechanics, exposure, and specimen extraction [2, 3, 6, 7]. The growing diversity of the platform landscape also reflects pronounced geographic diversification: systems have been developed and evaluated predominantly in Japan (hinotori), China (KANGDUO, SHURUI, Toumai, EDGE, CARINA), the United Kingdom (Versius), Switzerland (Dexter), France (Maestro), and Israel (Anovo), alongside the established US-based ecosystem.

The resulting evidence base is challenging to interpret because it is dominated by first-in-human reports, pilot studies, feasibility series, retrospective single-center cohorts, and only a limited number of comparative, economic, or learning-curve analyses [4, 7–11]. Existing reviews have largely addressed single-site robotic surgery, individual platforms, or single indications such as deep infiltrating endometriosis, rather than providing a comprehensive platform-by-platform synthesis of emerging robotic systems across the gynecologic spectrum [1, 2, 4, 7, 8, 10, 11].

In this review, the term emerging robotic platforms was used pragmatically to denote robotic systems that are newer to gynecologic surgical practice or that embody alternative technological configurations relative to conventional multiport robotic surgery. The term therefore does not imply uniformly early clinical evidence; rather, the defining criterion is technological differentiation from conventional multiport robotic surgery and/or recent entry into gynecologic practice, not uniformly early clinical adoption. This heterogeneity can be interpreted through the IDEAL framework and its device-focused extension IDEAL-D, which describe progression from idea and development to exploration, assessment, and long-term study [12–14]. Accordingly, some systems in the present review remain represented mainly by developmental or exploratory studies, whereas others - notably da Vinci SP, HUGO, and Senhance - already include more mature comparative, registry, or learning-curve evidence. Unlike prior single-platform or single-indication reviews, the present synthesis maps the entire emerging robotic ecosystem in gynecologic surgery, compares the maturity of evidence across platforms, and identifies gaps in cost, learning-curve, and long-term outcome reporting. Given this scope, the objective of this systematic review was to provide a comprehensive platform-by-platform synthesis of the available clinical evidence on 13 emerging robotic systems in gynecologic surgery, organized by procedure and encompassing surgical outcomes, economic data, and adoption trajectories.

## Methods

### Protocol registration and reporting framework

This review was prospectively registered in PROSPERO (CRD420261360389) and is reported in accordance with the PRISMA 2020 guidelines. The review question was formulated using a PICO framework.

### Review question and eligibility framework

The review question was to synthesize the current evidence on emerging robotic platforms in gynecologic surgery, structured according to the following PICO framework. Population: patients undergoing gynecologic surgery; Intervention/Exposure: da Vinci SP, HUGO, Senhance, Versius, hinotori, Dexter, SHURUI, Toumai, KANGDUO, EDGE, Anovo/Hominis, Maestro, or CARINA; Comparator: any comparator or none; Outcomes: surgical outcomes, cost, learning curve; Study designs: any clinical study type. Non-gynecology-focused multispecialty studies were retained when at least one eligible gynecologic procedure was explicitly reported; however, outcome data were extracted only when gynecology-specific or procedure-specific values were separately available. Studies evaluating non-eligible platforms, procedure types, or non-original article types were excluded. Case reports and very small case series were excluded when they duplicated procedures and platforms already represented by larger clinical series; they were retained only when they provided the only available human gynecologic evidence for a given platform or access strategy. Platforms with very limited gynecologic data (Maestro, Toumai, CARINA, EDGE, Anovo/Hominis) were nonetheless retained because they represent distinct technological trajectories - AI-assisted surgical assistance, telesurgery, modular systems, dedicated single-port access, or transvaginal robotic NOTES - whose inclusion was essential for a complete mapping of the emerging landscape.

### Information sources and search strategy

Electronic searches were conducted on 4 April 2026 in PubMed (*n* = 224), Scopus (*n* = 320), and Web of Science (*n* = 349), yielding 893 records before de-duplication. The search combined terms for emerging robotic platforms, gynecologic procedures, and robotic surgery methodology. The full search strategy is provided in Supplementary Appendix S1.

### Study selection

**Fig. 1:**
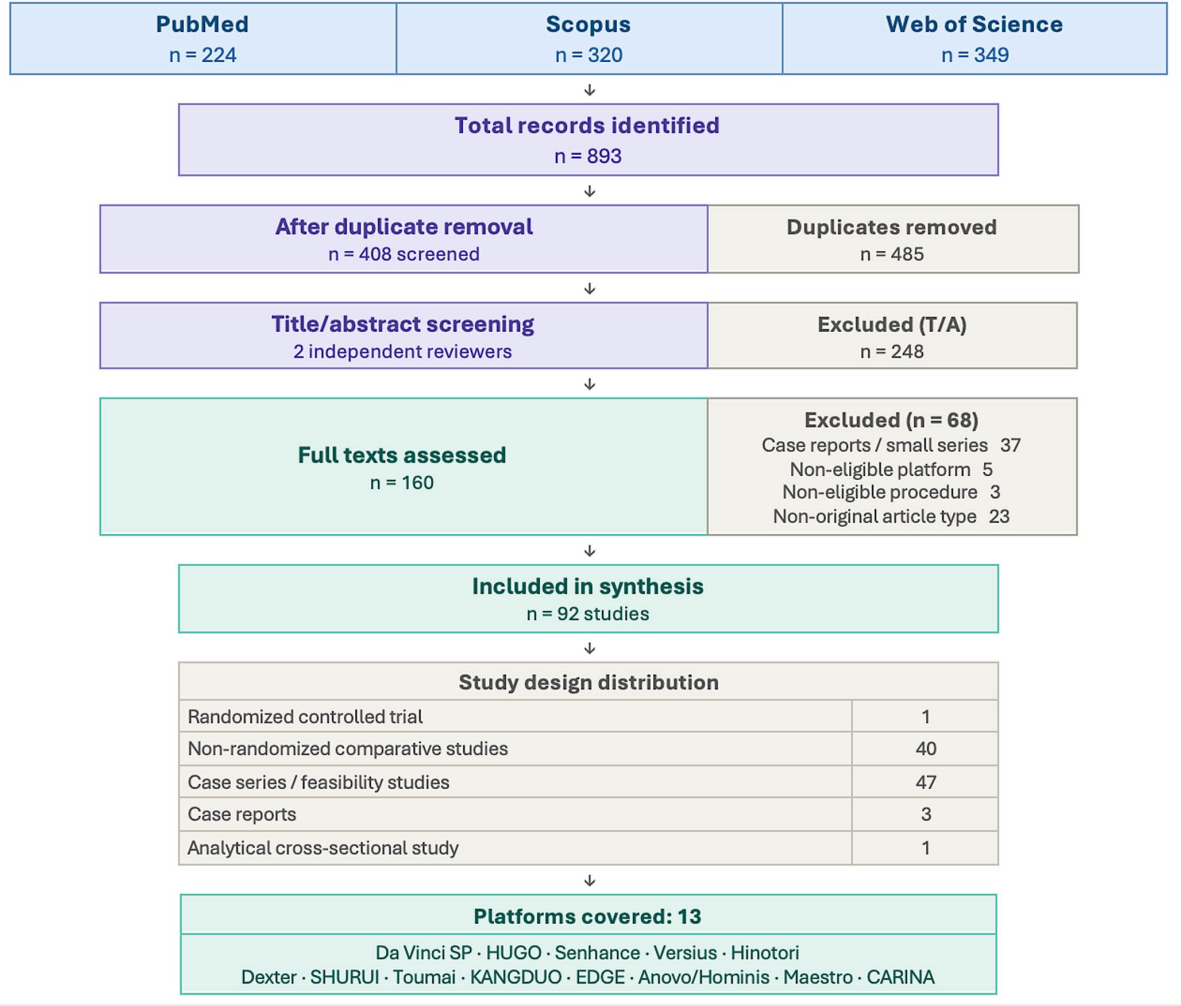
PRISMA 2020 flow diagram of study selection. Records were identified through PubMed, Scopus, and Web of Science, screened after duplicate removal, assessed for full-text eligibility, and included in the final qualitative synthesis. The review was registered in PROSPERO (CRD420261360389)

After removal of 485 duplicates, 408 unique records underwent title/abstract screening independently by two reviewers; disagreements were resolved by discussion and consensus. Of these, 248 were excluded and 160 full-text articles were retrieved for eligibility assessment. 68 full-text articles were excluded for one primary reason each: 37 case reports or small case series, 5 non-eligible platforms, 3 non-eligible procedure types, and 23 non-original article types. 92 studies met all eligibility criteria and were included in the final synthesis (Fig. 1).

### Data collection process

Data were extracted independently from the full texts of all 92 included studies by the same two reviewers (M.B., D.A.) who conducted the screening; disagreements were resolved by consensus. Extraction focused on platform, study design, country, procedure type, sample size, comparator, operative time, console time, docking time, estimated blood loss, complications (categorized by Clavien-Dindo grade), conversion rate, hospital stay, cost information, and learning-curve data. Only explicitly stated data were extracted; missing values were recorded as not reported rather than imputed.

### Outcomes and variables of interest

Primary domains: (1) type of gynecologic procedure and surgical outcomes; (2) cost data; (3) learning-curve data. Secondary variables: study design, sample size, country, and comparator characteristics.

### Risk of bias assessment

Risk of bias was assessed independently by two reviewers using a design-appropriate instrument for each included study; disagreements were resolved by consensus. The single randomized trial was assessed with RoB 2. Non-randomized comparative studies were assessed with the JBI Cohort Checklist. Single-arm case series and feasibility studies were assessed with the JBI Case Series Checklist, case reports with the JBI Case Report Checklist, and one learning-curve study with a cross-sectional design with the JBI Analytical Cross-Sectional Checklist. Full item-level assessments and overall concern judgments are reported in Supplementary Appendix S2.

### Data synthesis and additional analyses

Meta-analysis was not planned or performed, as pre-specified in the registered protocol. Statistical synthesis was not appropriate given the distribution of included studies across 13 distinct robotic platforms, multiple procedure types and study designs, combined with inconsistent outcome definitions - particularly for operative time components, complication grading, and economic variables - and the absence of more than one randomized trial. Observed differences in operative time, blood loss, docking time, and length of stay across platforms cannot be interpreted as platform-intrinsic advantages, because they are strongly confounded by case mix, surgeon experience, institutional workflow, adoption phase, and reporting conventions. Cross-platform ranking on aggregate outcome metrics was therefore not attempted.

## Results

### Study characteristics

The 92 included studies covered 13 robotic platforms and were weighted toward observational evidence: one randomized trial, 40 non-randomized comparative studies, 47 case series or feasibility studies, three case reports, and one analytical cross-sectional learning-curve study. Evidence density was highly uneven across platforms, with the largest bodies of literature concentrated in da Vinci SP and HUGO. Detailed platform characteristics are summarized in Table 1.

**Table 1:** Characteristics of emerging robotic surgical platforms in gynecologic surgery

Platform	Manufacturer	Origin	First regulatory approval	Console type	Arm architecture	Port access strategy	Gynecologic procedures reported	Studies (*n*)*	Study designs
Da Vinci SP	Intuitive Surgical	USA	2014 (FDA)	Closed	Single-port platform: 3 instrument arms + 1 articulating camera	Single-port	Hysterectomy, myomectomy, EC staging + SLN, radical hysterectomy, trachelectomy, oophorectomy, sacrocolpopexy, ovarian cancer staging, vNOTES	29	Retrospective cohorts, comparative cohorts with PSM, multi-institutional studies
HUGO RAS	Medtronic	USA	2021 (CE)	Open	Modular multi-arm: up to 4 independent arms	Multi-port	Hysterectomy, EC staging, adnexal surgery, sacrocolpopexy, endometriosis, myomectomy, pelvic floor surgery	22	Prospective and retrospective cohorts, comparative cohorts with PSM, structured learning-curve studies
Senhance	Asensus Surgical	USA	2017 (FDA)	Open	Multi-arm: 3 independent arms	Multi-port	Hysterectomy (incl. obese), EC staging, sacrocolpopexy, oncology (multi-disciplinary)	9	Prospective registry (TRUST), retrospective cohorts, cost analyses, FLS simulation
Versius	CMR Surgical	UK	2019 (CE)	Open	Modular multi-arm: up to 4 independent arms	Multi-port	Hysterectomy, radical hysterectomy, EC staging + SLN, sacrocolpopexy, adnexectomy	8	Prospective cohort (IDEAL 2b), registry, retrospective cohorts
Hinotori	Medicaroid	Japan	2020 (PMDA)	Closed	Boom-mounted multi-arm: 4 robotic arms	Multi-port	Hysterectomy, EC staging + SLN, sacrocolpopexy	8	Retrospective comparative cohorts, case series
Dexter	Distalmotion	Switzerland	2020 (CE)	Open (hybrid sterile)	Hybrid modular: 2 robotic bedside arms on demand	Multi-port (hybrid)	Hysterectomy, endometriosis, sacrocolpopexy	4	Prospective multicenter studies, retrospective studies, case report
SHURUI (SR-ENS-600)	Beijing Surgerii Robotics	China	2023 (NMPA)	Closed	Single-port platform: 4 bendable snake-shaped arms	Single-port	Hysterectomy, myomectomy, ovarian cystectomy, EC/cervical staging	3	IDEAL 2a study, prospective multicenter trial, retrospective cohort
Toumai	MicroPort MedBot	China	2022 (NMPA)	Closed	Boom-mounted multi-arm: 4 robotic arms	Multi-port	Hysterectomy	3	Case report, pilot study, multispecialty retrospective series
KANGDUO (KD-SR-01)	Sagebot/Kangduo Robot Co.	China	2022 (NMPA)	Closed	Boom-mounted multi-arm: 4 robotic arms	Multi-port	Hysterectomy, ovarian cystectomy, adnexectomy, EC staging, radical hysterectomy	3	Randomized non-inferiority trial, retrospective comparative studies
EDGE SP1000	Shenzhen Edge Medical Device	China	2023 (NMPA)	Closed	Single-arm single-port platform	Single-port	Hysterectomy, gynecologic oncology staging	2	Prospective single-arm trial, pilot case series
Anovo/Hominis	Momentis Surgical	Israel	2021 (FDA)	Open	Transvaginal: 2 humanoid-shaped robotic arms	RvNOTES	Hysterectomy, adnexectomy	2	Prospective 2-center studies
Maestro	Moon Surgical	France	2022 (FDA)	AI-assisted solo	Assistive: 2 articulated co-manipulated arms	Multi-port	Sacrocolpopexy	1	IDEAL 2a prospective multicenter study
CARINA	Ronovo Surgical	China	2025 (NMPA)	Closed	Modular multi-arm: 4 independent arms	Multi-port	Radical hysterectomy, hysterectomy	1	Retrospective case series

AI = artificial intelligence; CE = Conformité Européenne; EC = endometrial cancer; FDA = US Food and Drug Administration; FLS = Fundamentals of Laparoscopic Surgery; IDEAL = Idea, Development, Exploration, Assessment, Long-term study framework; NMPA = National Medical Products Administration (China); PMDA = Pharmaceuticals and Medical Devices Agency (Japan); PSM = propensity score matching; RAS = robot-assisted surgery; RCT = randomized controlled trial; SLN = sentinel lymph node; vNOTES/RvNOTES = (robotic) vaginal natural orifice transluminal endoscopic surgery. *Counts refer to platform-attributed unique studies included in the review. In Tables 2, 3, 4, 5, the same study may appear in more than one procedure block when separate procedure-specific data were extractable; therefore, table rows do not necessarily equal the number of unique studies.

**Fig. 2:**
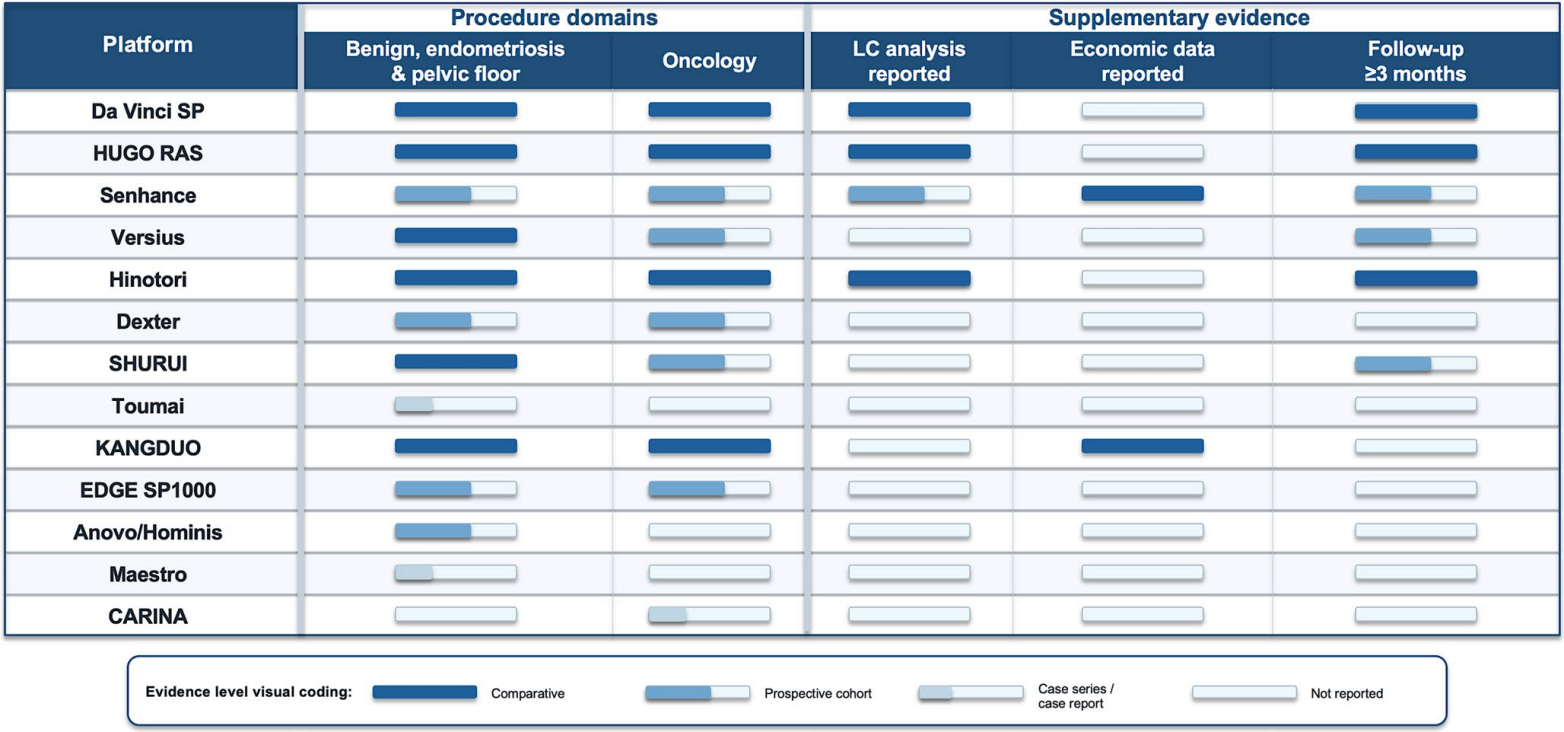
Platform-level evidence map across emerging robotic systems in gynecologic surgery. The figure summarizes the level and availability of evidence across platforms and is intended as a broad visual overview; detailed data are reported in the Results sections and in Tables 2, 3, 4, 5. For the combined benign/endometriosis/pelvic floor domain, the visual code reflects the highest level of evidence identified across categories. Comparative study includes randomized, propensity-score matched, or other comparative clinical studies. Prospective cohort includes single-arm prospective cohorts, registries, or IDEAL-stage studies without comparator. Case series/case report includes retrospective series, feasibility reports, pilot studies, and individual case reports. Learning-curve evidence includes structured analyses as well as indirect metrics. Economic data include reported cost metric or comparison. Follow-up was considered present when at least one included study reported outcomes at or beyond 3 months.

Figure 2 provides a concise platform-level evidence map, summarizing the highest level of evidence available across procedure domains, learning-curve analyses, economic data, and follow-up reporting.

Because the evidence base is dominated by retrospective single-center designs with predominantly moderate risk of bias, the findings reported below should be interpreted as evidence of technical feasibility and short-term safety rather than comparative efficacy or superiority.

### Da Vinci SP

The da Vinci SP platform contributed 29 platform-attributed studies. The platform was applied across a broad procedural range, including total and supracervical hysterectomy for benign disease, myomectomy, sacrocolpopexy and other prolapse procedures, endometrial cancer staging, early-stage endometrial cancer hysterectomy, selected ovarian cancer staging, radical hysterectomy, radical trachelectomy, ovarian cystectomy, and transvaginal robotic vNOTES procedures. Large early series suggested that a broad case mix could be completed through a single 2.5-cm umbilical incision with low conversion rates and limited blood loss [15–17]. In Kwak’s 100-case single-surgeon experience, a broad procedural mix-including myomectomy, endometrial cancer staging, radical hysterectomy, and radical trachelectomy was completed through a single 2.5-cm incision without conversion to multiport or open surgery and with limited blood loss [15].

For benign hysterectomy, the comparative data were generally favorable but not uniform. Against conventional single-port laparoscopy, single-port robotic hysterectomy showed lower blood loss and shorter operative time in some series [18], and comparable or better perioperative outcomes in others [19]. In contrast, supracervical hysterectomy for fibroids with da Vinci SP remained associated with longer operative time than conventional single-site laparoscopy, while other short-term outcomes were similar [20]. In propensity-matched comparisons against da Vinci Xi, perioperative outcomes were broadly comparable, though SP generally required longer operative and console times during the adoption phase; learning-curve analysis indicated proficiency achievable after approximately eight cases in surgeons with prior robotic experience [21–23].

Myomectomy data support feasibility but highlight case-selection effects. SP myomectomy achieved comparable short-term outcomes to conventional single-site robotic approaches in propensity-matched studies, but one comparison found lower estimated blood loss and shorter morcellation and total operative times with the coaxial Xi single-site approach than with SP [24]. A 216-case fertility-focused series demonstrated safe fertility preservation outcomes comparable to da Vinci Xi single-site [25], and a pilot series of 61 cases confirmed safety without conversion [26]. The comparison of SP versus reduced-port (1 + 1) approaches for myomectomy and hysterectomy found SP associated with lower blood loss in both procedures [27].

In the largest comparative cohort, SP sacrocolpopexy was associated with a 40-minute shorter operative time than multiport robotic sacrocolpopexy (176 vs. 216 min) and a shorter hospital stay, with similar blood loss, complication rates, and short-term recurrence outcomes [28]. An earlier comparison with da Vinci Xi single-site found equivalent operative times, blood loss, length of stay, and cystotomy rates [29]. A two-center series confirmed feasibility with a low conversion rate when sacrocolpopexy was combined with hysterectomy [30].

Gynecologic oncology results for da Vinci SP were generally reassuring though based mainly on short-term perioperative outcomes. Propensity-matched and retrospective series in early-stage endometrial cancer found similar blood loss, length of stay, complication rates, and short-term oncologic feasibility for SP and multiport robotic staging, with either similar or slightly shorter docking times for SP and broadly comparable total operative times [31–34]. In a low-risk endometrial cancer pilot study, 25 patients underwent SP hysterectomy through a single 2.5-cm incision with favorable intraoperative outcomes and no major safety concerns [35]. Preliminary ovarian cancer staging in seven patients was completed without conversion or intraoperative complications [36].

Transvaginal natural orifice approaches represent a further dimension of the da Vinci SP evidence base. A propensity-matched comparison found lower blood loss with SP robotic versus conventional laparoscopic vNOTES [37], and a 28-case RSP-vNOTES series documented a 3.6% conversion rate and 7.1% Clavien-Dindo ≥ II rate, consistent with early-adoption expectations [38]. A hostile-abdomen case demonstrated single-port robotic transvaginal hysterectomy feasibility in complex pelvic conditions [39]. Cost data for da Vinci SP in gynecology were sparse: no included study provided a formal platform-specific cost analysis. Learning-curve evidence was stronger than for most competing platforms: a 2025 CUSUM analysis demonstrated mastery after 13 SP hysterectomy cases versus 50 multiport cases, with a significantly smaller hemoglobin drop in the SP cohort [40], and additional studies supported proficiency after approximately eight cases for benign hysterectomy [21] and fewer than 15 for single-incision sacrocolpopexy [29].

### HUGO

HUGO was the second most extensively studied platform, with 22 reports. The earliest larger European experience from Gemelli Hospital included 138 women for uterine/adnexal disease or prolapse, demonstrating efficient docking and console times with low rates of redocking or laparoscopic completion [41]. Subsequent hysterectomy series across different settings confirmed safety and feasibility: a European pilot reported a median operative time of 127 min with no intraoperative complications [42]; real-world data from Panama showed shorter operative time and lower blood loss than conventional laparoscopy (76 vs. 117 min; 30 vs. 200 mL) [43]; a single-center Indian series documented no conversions [44].

Sacrocolpopexy is the reconstructive indication best documented for HUGO. The first dedicated 60-case series reported high anatomical and subjective success rates at 3 months (96.7% and 98.3%) with efficient docking and console times (median of 4 and 134 min) [45]. Both head-to-head comparison with da Vinci Xi and propensity-matched comparison with laparoscopy demonstrated equivalent operative times, complication rates, and short- to mid-term functional outcomes [46, 47]. A series from a robotics-naive centre confirmed feasibility but documented substantially longer operative time and higher blood loss, with 25% grade I–II complications, underscoring the importance of learning effects and case complexity [48].

Endometriosis surgery appears feasible on HUGO, although the current literature remains early-phase. Initial series were completed without conversion, with minor postoperative events and - in an Italian tertiary-center series - one bladder laceration alongside substantial short-term improvements in dysmenorrhea, dyspareunia, dyschezia, and chronic pelvic pain [49, 50]. The first multicenter comparative study between da Vinci and HUGO for deep endometriosis found similar operative time and blood loss between platforms, with no clear disadvantage for HUGO, although patient-reported functional improvement favored da Vinci in that specific cohort [51].

Cost evidence for HUGO in gynecology remains underdeveloped. None of the gynecology-focused HUGO studies offered a formal cost-effectiveness or hospital-cost comparison. Learning-curve evidence is one of the platform’s comparative strengths. CUSUM analysis of docking time across the first 192 gynecologic cases demonstrated efficient team learning comparable to established platforms [52], and a 12-month real-world experience confirmed rapid adaptation across port placement, docking, and console work for hysterectomy and endometriosis without conversion [53]. Hysterectomy-focused learning data with the narrow-setting arm configuration showed docking proficiency achievable after approximately 19 cases [54]. In the direct comparison between a da Vinci-experienced and a robotics-naive surgeon, the experienced operator stabilized by case 5, whereas the novice required approximately 15 cases to achieve a comparable operative-time plateau, without safety compromise [55].

### Senhance

Senhance contributed nine studies. The gynecology-focused Senhance literature covers total hysterectomy, hysterectomy in obese patients, sacrocolpopexy, and mixed first-100-case institutional experiences, while one simulation study, one large cross-specialty safety registry, and one oncology feasibility cohort added contextual information on learning and safety. An early pilot study in obese women (*n* = 10, BMI 30–40) confirmed feasibility without conversion [56], and more mature multicenter data from 295 cases demonstrated a 1% conversion rate, 2% adverse event rate, and competitive operative times reflecting a post-adoption phase [57].

Reconstructive data are also meaningful. In the 25-case sacrocolpopexy series, no major intraoperative complications and no subjective recurrences occurred, though two asymptomatic anatomical recurrences and two 30-day readmissions for bowel obstruction were recorded [58]; a statistically significant reduction in operative time across the series (231.7 vs. 190.3 min; *p* < 0.05) provided an early learning-curve signal. The safety registry TRUST, enrolling 3,239 patients including 609 gynecologic procedures, reported a Clavien-Dindo ≥ II rate of 3.9% and a conversion rate of 4.5% for the overall registry cohort [59], while an oncology feasibility analysis from the same registry confirmed acceptable safety across 966 multidisciplinary oncologic procedures [60]. Cost evidence is strongest for Senhance among all platforms in this review. Instrument costs were substantially lower than with da Vinci ($559 vs. $1,393; *p* < 0.001) while console times were comparable, and Senhance instrument costs were similar to laparoscopic-assisted vaginal hysterectomy ($559 vs. $498) [61]. In robotic sacrocolpopexy, after adjustment for operative time and blood loss, Senhance was associated with a significantly lower total hospital-system cost by $908.33 [62]. The simulation study showed that residents, fellows, and attending surgeons all adapted rapidly to Senhance haptic controls, with measurable improvement over repeated attempts and no detriment from force-feedback activation [63].

### Versius

Eight studies addressed Versius, covering total laparoscopic hysterectomy, hysterectomy for benign and malignant disease, radical hysterectomy, sacrocolpopexy, early-stage endometrial cancer staging with sentinel node biopsy, a pelvic surgery registry, and one cross-platform anesthesiologic comparison. In the IDEAL-D stage 2b cohort (*n* = 144, three hospitals), the unplanned conversion rate was 1.4%, adverse events occurred in fewer than 5%, and one device-related serious adverse event was recorded [64]. A propensity-matched Polish comparison confirmed longer operative time with Versius than with laparoscopy (134.6 vs. 96.5 min; *p* < 0.01) while blood loss, complications, and length of stay did not differ [65], identifying operative time as the primary trade-off rather than a safety concern.

Oncologic experience is encouraging. An early radical hysterectomy series (*n* = 30) reported a mean lymph node yield of 24.7 and two ureterovaginal fistulas requiring postoperative management [66]. Hysterectomy with sentinel node biopsy for early endometrial cancer (*n* = 14) was completed without conversion or complications, achieving 100% sentinel node detection without readmissions or reoperations [67]. In a prospective single-center sacrocolpopexy series, 20 women underwent nerve-sparing robotic sacrocolpopexy; all procedures were completed without complications or conversions, and 90% achieved complete anatomical correction at 3 months with parallel improvement in patient-reported outcomes [68]. A multi-center pelvic surgery series (42 procedures, 11 gynecologic) confirmed feasibility without intraoperative conversion, noting occasional system alarms and the need to adjust trocar placement according to patient height [69]. The Versius Surgical Registry provided broader safety reassurance across 2,083 cases including a large gynecology component [70]. No gynecology-specific cost or formal learning-curve data were identified.

### Hinotori

The hinotori platform was represented by eight studies, nearly all from Japan, with evidence centered on hysterectomy, endometrial cancer surgery with sentinel node biopsy, sacrocolpopexy, and cross-platform analyses. The first report described 12 hysterectomies for a mixed case mix including endometrial cancer, fibroids, atypical hyperplasia, adenosarcoma, and high-grade cervical intraepithelial neoplasia. Median console and total operative times were 161 and 214 min, with no conversion and one postoperative pelvic infection [71]. Subsequent analyses confirmed feasibility but documented that time metrics remain somewhat longer than those of more established platforms during early adoption.

The most informative oncologic comparison evaluated simple hysterectomy with sentinel lymph node biopsy for low-risk endometrial cancer. Operative and console times were similar between hinotori and da Vinci Xi, but the pre-console interval was longer with hinotori (*p* = 0.004), suggesting additional setup demands without a clear disadvantage in core surgical execution [72]. A larger retrospective comparison across 401 cases found no significant differences in total operative time, blood loss, or length of stay, though console time remained marginally longer for hinotori [73]. The four-system comparison with da Vinci X, da Vinci Xi, HUGO, and hinotori confirmed safe hysterectomy performance across all included platforms with only small early-case differences in setup time [74]. Reconstructive data on sacrocolpopexy are also reassuring. Two comparative series against da Vinci found consistently longer operative and console times with hinotori, while lower urinary tract symptom scores, recurrence outcomes, complications, and hospital stay were comparable across both studies [75, 76]. A single-surgeon series specifically analyzed suture performance and found a longer mean stitch time with hinotori (76 vs. 60 s per stitch; *p* < 0.005) [77]. No formal gynecology-specific cost studies or dedicated CUSUM learning-curve analyses were identified. Hinotori should currently be viewed as clinically viable with growing comparative evidence, still warranting dedicated investigation.

### Dexter

Dexter contributed four gynecology-focused studies, all centered on hysterectomy. The first robot-assisted hysterectomy with Dexter was completed without intraoperative complications, with estimated blood loss of 10 mL, operative time of 150 min, console time of 120 min, and total docking time of 6 min [78]. A 52-patient multicenter single-arm study, and a 34-patient prospective multicenter series, procedures were completed without device-related adverse events; two Clavien-Dindo IIIb complications were recorded in the larger series (neither device-related), and three cases were completed laparoscopically by surgeon choice [79, 80]. A retrospective series illustrated integration into a hybrid laparoscopic-robotic workflow, combining robotic and conventional steps according to procedural need - a feature consistently highlighted as a potential infrastructure advantage for lower-volume centres [81]. No cost study or formal learning-curve analysis was identified.

### SHURUI

Three studies examined SHURUI, all focused on single-site gynecologic surgery. In the IDEAL stage 2a first-in-human trial, 10 women with progressively more complex gynecologic indications underwent surgery without conversion or serious complications at 3-month follow-up [82]. A multicenter prospective clinical trial expanded the evidence to 63 women treated across six academic centers for ovarian cysts, myomas, cervical epithelial neoplasia, and endometrial cancer. Average operative time was 157.0 min, estimated blood loss 63.9 mL, and average hospital stay 3.63 days; there were no conversions, complications, or readmissions [83]. The most informative comparative study found SHURUI associated with lower intraoperative blood loss and shorter time to flatus than both da Vinci single-site and multiport controls, with no complications at 3-month follow-up [84]. No cost or learning-curve data were identified.

### Toumai

The Toumai literature comprised three records, all focused on telesurgery. The most informative was the first European telesurgical hysterectomy, performed remotely between institutions approximately 20 km apart and completed without intraoperative complications; total operative time was 74 min, mean network latency 20 ms, and discharge occurred on postoperative day 2 without complications at 18-day follow-up [85]. A pilot investigation of robot-assisted total hysterectomy with Toumai in Europe supports the same direction of evidence but contributes limited extractable outcome information [86]. A multi-center multispecialty telesurgery series of 66 Toumai procedures across China included one gynecologic case and no conversions to local surgery, with a gynecology-specific network delay of approximately 61 ms [87]. No cost or learning-curve data were available.

### KANGDUO

The KANGDUO platform was represented by three studies that together constitute an unusually informative early evidence package: a broad mixed gynecology series, a multicenter randomized noninferiority trial, and a retrospective comparative study in endometrial cancer. In the broadest report, the KD-SR-01 system was used for 242 gynecologic procedures including 144 hysterectomies for benign uterine tumors, 25 ovarian cystectomies, 25 unilateral adnexectomies, 24 staging procedures for early endometrial cancer, and 24 radical resections for early cervical cancer. No patient experienced a serious Clavien-Dindo grade ≥ III complication. Compared with conventional laparoscopy, KANGDUO surgery was associated with shorter hospitalization, shorter operative time, lower blood loss, and lower drainage volume, while the complication rate remained similar [88]. This is one of the few studies in this review reporting more favorable short-term perioperative outcomes than laparoscopy across a broad mixed gynecology case mix.

The endometrial cancer data are especially important. Procedures were completed without conversion, the primary noninferiority criterion for lymph node dissection was met, and perioperative outcomes including blood loss and complications were comparable; docking time was marginally longer with KANGDUO [89]. This trial provides the strongest direct comparative evidence for any Chinese platform in this review. The retrospective Harbin study of 211 stage T1 endometrial cancer patients further supported comparable efficacy and safety: surgical success was 100% in both groups, blood loss and complication rates were similar, but the KANGDUO group had longer operative time, console time, time to first flatus, and hospital stay than the da Vinci group [90]. Cost evidence was also notable: both total hospitalization costs and surgical expenses were significantly lower with KANGDUO than with da Vinci, despite the longer time metrics [90]. Overall, KANGDUO stands out for combining broad case-mix feasibility, randomized comparative data, and an initial economic advantage signal within its regulatory context.

### EDGE

Two studies evaluated the EDGE SP1000 single-port platform. The first pilot series included 33 women with benign or malignant gynecologic disease; mean operative time was 105.5 min, mean estimated blood loss 34.6 mL, no conversions occurred, one patient developed postoperative fever, and pain scores and scar healing were satisfactory [91]. The second, a prospective single-arm trial in 18 women (8 malignant), showed mean operative times of 190.1 min for benign and 254.4 min for malignant cases, mean blood loss of 25 mL, no assistant ports or conversions, no perioperative complications, and high cosmetic satisfaction with the umbilical wound at 1 month [92]. No cost studies or formal learning-curve analyses were identified. EDGE should currently be regarded as an early single-port system with encouraging feasibility and cosmetic results, with evidence still confined to first-institution experiences.

### Anovo

The Anovo Surgical System (formerly Hominis; Momentis Surgical, Tel Aviv, Israel - previously Memic Innovative Surgery) is distinct from the other systems in this review because it was purpose-built for robotic vaginal natural orifice transluminal endoscopic surgery (RvNOTES). Two prospective two-center studies were included. In the earlier bilateral salpingo-oophorectomy series, eight women with nonmalignant indications were treated successfully without conversion; median procedure duration 45 min, visual analog pain score 1 at 24 h, procedure duration decreased over the study period, and surgeon usability scores were 5/5 [93]. The later RvNOTES hysterectomy series of 30 women with benign indications showed excellent feasibility: all procedures were completed without conversion; there were no intraoperative complications; median procedure time 57 min; postoperative pain was minimal; median hospital stay was 3 days; and complete vaginal cuff healing was confirmed at 6 weeks [94]. No cost studies were identified. Anovo should be interpreted as a concept-specific platform with promising data for transvaginal surgery; its principal strengths are low blood loss, low postoperative pain, and a scarless access strategy, while its main limitation is the very small evidence base.

### Maestro

Only one study was included. The IDEAL 2a prospective multicenter observational study evaluated the Maestro system in 45 procedures performed by five surgeons mainly across general surgery indications (cholecystectomy, inguinal hernia repair, sleeve gastrectomy), including 2 sacrocolpopexy procedures. Mean robotic setup time was 6.1 min, mean operative time 43.6 min, one conversion to multiport laparoscopy occurred, and no device-related adverse events were recorded [95]. No gynecology-specific outcome data were separately extractable. Therefore, Maestro currently provides only contextual feasibility evidence for gynecologic surgery and cannot yet be meaningfully evaluated as a gynecologic robotic platform on procedure-specific outcomes. Nevertheless, it was retained in this review because the included multispecialty cohort explicitly reported sacrocolpopexy procedures and because Maestro represents the first AI-enabled surgical assistant capable of physical interaction with the operative field, marking an important step toward more autonomous and digitally assisted surgical workflows.

### CARINA

CARINA was represented by a single retrospective case series of 16 women with gynecologic malignancies (10 cervical cancer, 6 endometrial cancer), all completed without conversion to laparoscopy or laparotomy. Mean docking time was 5.75 min. In the cervical cancer group, mean console and operative times were 154.6 and 211.9 min; in the endometrial cancer group, 98.7 and 153.3 min. Median estimated blood loss was 30 mL for cervical cancer and 20 mL for endometrial cancer, and no intraoperative or postoperative device-related complications were recorded [96]. No cost data or formal learning-curve analyses were reported. CARINA currently has only proof-of-feasibility evidence in gynecologic oncology, but that signal is noteworthy because it derives from complex malignant surgery rather than low-complexity benign procedures.

### Risk of bias within included studies

Among the 40 cohort studies, 9 were judged low concern, 30 moderate concern, and 1 high concern. Among the 47 case series, 7 were judged low concern, and 40 moderate concern. All three case reports were moderate concern. The single randomized trial raised some concerns under RoB 2. The single cross-sectional study was judged to have moderate concern. Taken together, the evidence base is dominated by retrospective single-center observational designs with predominantly low-to-moderate methodological rigor. The most consistent limitations across the included studies are the absence of concurrent control groups, incomplete confounder adjustment, short and incompletely reported follow-up, and inconsistent outcome measurement and reporting conventions. Full item-level assessments are provided in Supplementary Appendix S2.

### Platform- and study-level summary tables

To facilitate interpretation of the heterogeneous evidence base, Tables 2, 3, 4, 5 provide a platform- and study-level summary of surgical outcomes, conversion and complication rates, and available cost and learning-curve data.

**Table 2:** Da Vinci SP - surgical outcomes by procedure: per-study data

First author, year	Study design	Country	*N*	OR time (min)	Docking (min)	Console (min)	EBL (mL)	LOS (days)	FU	Conv. (%)	CD ≥ II (%)	Comparator/Notes
Hysterectomy (Benign)												
Kwak et al., 2022	Retrospective case series	South Korea	100 (mixed)	250 (median)	5.0	107.5	50	2.8	NR	0%	0%	Mixed cohort (76 myomectomies, 14 EC stagings, 3 radical hyst., 3 trachelectomies); cohort-level values shown
Shin et al., 2020	Retrospective cohort	South Korea	31 (mixed)	126.3 (mean)	2.2	NR	93.9	4.6	NR	0%	0%	Mixed cohort (hyst. + other); cohort-level values shown
Lee et al., 2023	Retrospective comparative cohort	South Korea	31 SP vs. 48 laparoscopy	111.26 (mean)	NR	NR	NR	3.94	NR	0%	NR	Single-site laparoscopy (*n* = 48) comparator; OR time longer in SP robotic (111.26 vs. 95.19 min; *p* < 0.01); EBL ↓ with SP (directional); 2 surgical complications in SP group (grade not specified); no conversion
Miyamura et al., 2025	Retrospective comparative cohort	Japan	23 SP	199 (median)	NR	146	12	NR	NR	0%	NR	SP laparoscopy (*n* = 33) comparator; EBL ↓ (12 vs. 80 mL; *p* < 0.01) and OR time ↓ (199 vs. 239 min; *p* < 0.03) with SP robotic; pneumoperitoneal time 146 min (SP) vs. 186 min (laparoscopy)
Alwafai et al., 2025	Retrospective case series	Germany	62 (mixed)	136.2 (mean)	8.3	NR	NR	3.6	NR	0%	3.2%	Mixed cohort (benign + oncologic); German-speaking centers
Park et al., 2023	Retrospective comparative cohort	South Korea	67 SP hysterectomy	NR	1.7	NR	97.4	NR	NR	0%	NR	da Vinci Xi (multiport) comparator
Higuchi et al., 2025	Retrospective comparative cohort with PSM	Japan	131 SP	94 (median)	2	66	5	4	NR	0%	3.1%	da Vinci Xi (multiport, *n* = 131)
Erdemoglu et al., 2025	Retrospective comparative cohort	USA	48 SP	178 (median)	NR	NR	NR	NR	NR	0%	NR	da Vinci Xi (multiport) comparator; CCI endpoint
Kanno et al., 2025	Retrospective comparative cohort with PSM	Japan	65 SP VANH	75.4 (mean)	2.9	97.3	94.5	4.0	NR	0%	4.6%	Laparoscopic VANH comparator; vNOTES-assisted
Guan et al., 2024	Case series	USA	28	188.7 (mean)	2.9	97.3	32.1	NR	NR	3.6%	7.1%	RSP-vNOTES hysterectomy; no comparator
Santillan-Gomez et al., 2024	Case report	USA	1	NR	NR	NR	NR	NR	NR	0%	0%	RSP-vNOTES in hostile abdomen; no comparator; feasibility in complex adhesion/prior surgery setting
Myomectomy												
Kwak et al., 2022	Retrospective case series	South Korea	76	250 (mixed)	5.0	107.5	50	2.8	NR	0%	0%	Myomectomy subset of 100-case mixed cohort
Lee et al., 2022	Prospective case series	South Korea	61	149.9 (mean)	NR	NR	NR	4.5	NR	0%	0%	SP robotic myomectomy pilot; no comparator
Lee et al., 2024	Retrospective comparative cohort	South Korea	94 SP robotic myomectomy	NR	NR	NR	NR	NR	NR	0%	NR	Reduced-port Xi comparator; SP vs. 1 + 1 approach
Kim et al., 2023	Retrospective comparative cohort	South Korea	56 SP robotic myomectomy	NR	NR	NR	NR	NR	NR	0%	NR	Single-port laparoscopy comparator; EBL ↓ with SP
Kim et al., 2023	Retrospective cohort	South Korea	216 SP robotic myomectomy	81.7 (mean)	NR	NR	NR	NR	NR	0%	0%	da Vinci Xi (single-site) comparator; fertility outcomes reported
Lee et al., 2025	Retrospective comparative cohort with PSM	South Korea	108 SP	148.7 (mean)	NR	NR	203.9	2.06	NR	0%	NR	da Vinci Xi (single-site) comparator; EBL ↑ in SP group (203.98 vs. 102.33 mL; *p* < 0.001); OR time ↑ in SP (148.69 vs. 91.22 min; *p* < 0.001); LOS ↓ in SP (2.06 vs. 4.07 days; *p* < 0.001)
Endometrial cancer												
Seon et al., 2023	Retrospective comparative cohort with PSM	South Korea	42 SP	225 (median)	NR	125	30	2	NR	0%	NR	da Vinci Xi (*n* = 126) comparator; similar outcomes (shorter LOS, lower pain score, longer operation times)
Vizza et al., 2025	Prospective comparative cohort with PSM	Italy	50 SP	120 (median)	NR	NR	NR	NR	NR	0%	12%	da Vinci Xi (*n* = 50) comparator; multi-institutional
Cucinella et al., 2025	Retrospective multi-institutional comparative cohort	Italy	97 SP	140 (median)	10	NR	40	3	NR	NR	NR	da Vinci Xi (*n* = 92) comparator; includes atypical endometrial hyperplasia; CD ≥ II 10.3% = combined cohort (SP + Xi); no difference in conversion rate
Matsuura et al., 2024	Retrospective comparative cohort	Japan	16 SP	NR	NR	NR	NR	NR	NR	0%	NR	da Vinci Xi comparator (3-system: 16 SP, 10 hinotori, 10 HUGO); OR time ↓ with SP and HUGO vs. hinotori (*p* < 0.05); OR time for SP relatively stable across cases; early institutional comparison
Vizza et al., 2025	Prospective case series	Italy	25	115 (median)	10	65	67	NR	NR	0%	0%	No comparator; low-risk EC
Vizza et al., 2025	Retrospective case-control study	Italy	25 SP hyst.	110 (median)	NR	NR	50	3.5	NR	0%	NR	da Vinci Xi (single-site, *n* = 50) comparator; early EC hysterectomy
Chiofalo et al., 2025	Retrospective case series	Italy	7	NR	NR	NR	10	3	NR	0%	0%	Epithelial ovarian cancer staging; no comparator; exploratory
Sacrocolpopexy												
Shin et al., 2020	Retrospective cohort	South Korea	7 (mixed)	126.3 (mixed)	2.2	NR	93.9	4.6	NR	0%	0%	SCP subset of mixed cohort; procedure-specific values not separately extractable
Oh et al., 2023	Retrospective learning-curve study	South Korea	66 SP	201.5 (median)	NR	NR	NR	NR	NR	1.5%	NR	da Vinci Xi (single-site, *n* = 57) comparator; LC analysis; proficiency < 15 cases
Ashmore et al., 2024	Retrospective 2-center cohort	USA	69	209 (median)	NR	NR	100	NR	NR	1.4%	NR	No comparator; SCP with concomitant hysterectomy
Ferrigni et al., 2025	Retrospective cohort	USA	72 SP	176 (median)	NR	NR	NR	0.46	NR	0%	5.6%	da Vinci (multiport, *n* = 109) comparator; 40-min shorter OR time with SP
Other/learning curve												
Kim et al., 2023	Retrospective comparative cohort	South Korea	35 SP hyst./108 SP combined procedure	NR	NR	NR	NR	NR	NR	0%	NR	Single-port laparoscopy comparator; mixed hyst. + oophorectomy; EBL ↓ with SP
Vizza et al., 2025	Retrospective learning-curve study (CUSUM)	Italy	74 SP	114 (mean)	NR	NR	NR	NR	NR	0%	NR	da Vinci (multiport historical cohort, *n* = 73); mastery at 13 SP vs. 50 MP cases; Hb drop ↓ with SP

Table 2 includes study entries across five procedure blocks. Console time is retained (71% NR overall) because it is a platform-defining metric distinguishing setup time from surgical execution, and it is well-reported in the comparative hysterectomy and EC staging blocks where cross-platform comparison is meaningful.

CC = case-control; CCI = Comprehensive Complication Index; CD ≥ II = Clavien-Dindo grade ≥ II; Conv. = conversion to laparoscopy or laparotomy; CUSUM = cumulative sum analysis; EBL = estimated blood loss; EC = endometrial cancer; FU = follow-up duration (months unless otherwise stated); Hb = hemoglobin; hyst. = hysterectomy; LC = learning curve; LOS = length of hospital stay; MP = multiport; NR = not reported in the source study; OR time = skin-to-skin operative time; PSM = propensity score matching; RSP-vNOTES = robotic single-port transvaginal natural orifice transluminal endoscopic surgery; SCP = sacrocolpopexy; SP = single-port; VANH = vaginal-assisted NOTES hysterectomy; vNOTES = vaginal natural orifice transluminal endoscopic surgery. Values are median or mean as reported. Numeric fields report only directly extractable absolute values. Mixed-procedure cohorts are explicitly labelled; the same study may appear in more than one procedure block when separate extractable data were reported per procedure. ↑/↓ = directional finding not amenable to numeric extraction

## Discussion

### Principal findings

This systematic review synthesized 92 studies covering 13 emerging robotic platforms in gynecologic surgery and identified three principal findings. First, technical feasibility with generally acceptable short-term perioperative safety was the most consistent conclusion across platforms, not comparative superiority. Most systems supported hysterectomy; selected centers extended use to myomectomy, sacrocolpopexy, endometriosis surgery, and limited oncologic procedures, typically with low conversion rates and modest blood loss. These findings arise predominantly from early adoption studies in expert centers and must not be interpreted as evidence that platforms are interchangeable or equivalent across indications and contexts. The predominantly moderate risk-of-bias profile of the included studies further limits the strength of any cross-platform inference.

**Table 3:** HUGO RAS - surgical outcomes by procedure: per-study data

First author, year	Study design	Country	*N* (HUGO)	OR time (min)	Docking (min)	Console (min)	EBL (mL)	LOS (days)	FU	Conv. (%)	CD ≥ II (%)	Comparator/Notes
Hysterectomy (Benign/malignant)												
Gioe et al., 2024	Prospective cohort	Italy	78 (uterine/adnexal)	NR	5	90	NR	NR	NR	2.6%	0%	First European HUGO experience; 138 total (including 60 POP)
Monterossi et al., 2023	Prospective pilot study	Italy	20	127 (median)	NR	NR	50	NR	NR	0%	5%	1 grade 2 postoperative complication; no intraoperative events
Vargas Castillo et al., 2025	Retrospective comparative cohort	Panama	63	76 (median)	NR	NR	30	NR	NR	0%	0%	laparoscopy (*n* = 59) comparator; OR time ↓ and EBL ↓ with HUGO
Collà Ruvolo et al., 2024	Prospective 2-center study	Italy/Belgium	20 hyst	108.5 (median)	8	52.5	NR	NR	NR	0%	0%	Two tertiary robotic centers; 32 total procedures (20 hyst, 7 adnexal, 5 pelvic floor)
Yagur et al., 2024	Retrospective case series	Israel	16 hyst (60 total)	NR	5.9	65	NR	NR	12 mo	0%	NR	Benign gynecology; 12-month experience; 60 total: 32 endometriosis (53%), 16 hysterectomies (27%), 12 other (20%); docking 5.9 min; console 65 min
Nagata et al., 2025	Retrospective comparative study, 4-system	Japan	NR HUGO	NR	NR	NR	NR	NR	NR	0%	NR	da Vinci X or Xi and hinotori comparators
Anagani et al., 2025	Retrospective single-center case series	India	20 gyn (18 hyst)	NR	6.3	86.9	103.5	2	NR	0%	NR	No comparator
Nozaki et al., 2025	Retrospective learning-curve study (CUSUM)	Japan	78	68 (median)	9	46	5	NR	NR	0%	NR	Narrow arm-setting; docking proficiency at 19 cases by CUSUM
Komatsu et al., 2026	Retrospective learning-curve study	Japan	43	NR	NR	NR	NR	NR	NR	0%	NR	Experienced vs. robotics-naive surgeon; stabilization at case 5 vs. case 15 respectively
Sacrocolpopexy/pelvic floor												
Panico et al., 2023	Prospective case series	Italy	60	185 (median)	4	134	15	NR	3 mo	0%	NR	First 60 HUGO SCP; 96.7% anatomical success at 3 mo; 98.3% subjective success
Ruvolo et al., 2024	Retrospective comparative cohort	Italy/Belgium	15 HUGO	120 (median)	NR	NR	NR	NR	NR	0%	0%	da Vinci Xi (*n* = 23) comparator; first head-to-head SCP comparison
Mastrovito et al., 2025	Retrospective comparative cohort with PSM	Italy	142 HUGO	NR	NR	NR	NR	NR	NR	0%	4.2% (HUGO)	laparoscopy (*n* = 142) comparator (PSM from 450 total); OR time ↑ and EBL ↓ with HUGO; CD ≥ II comparable (4.2% HUGO vs. 5.6% laparoscopy; p not significant); high-volume tertiary center
Albertus-Bofarull et al., 2026	Prospective case series	Spain	8	230 (median)	NR	NR	275	3	NR	0%	NR	No prior robotic experience; 25% grade I–II complications; demonstrates learning-curve effect
Panico et al., 2023	Retrospective learning-curve study (CUSUM)	Italy	192 (mixed)	NR	NR	NR	NR	NR	NR	NR	NR	Procedure-independent docking LC across entire gynecologic HUGO experience
Endometriosis surgery												
Olsen et al., 2024	Retrospective case series (IDEAL 1/2a)	Denmark	12	NR	17	87.5	40	1	NR	0%	0%	First HUGO endometriosis series; 4 grade I postoperative events; no intraoperative complications
Pavone et al., 2024	Retrospective case series	Italy/France	15	186.5 (median)	NR	NR	50	3	NR	0%	NR	1 bladder laceration; pain improvement at follow-up
Ianieri et al., 2024	Retrospective multicenter comparative study	Italy/France	16	NR	NR	NR	NR	NR	NR	0%	NR	da Vinci (multicenter); OR time mean 203.4 ± 68.5 min; no significant difference in OR time or EBL between platforms; CD: 4 events in da Vinci (20%) vs. 2 in HUGO (12.5%); functional improvement (dyspareunia, urinary, GI function) greater in da Vinci arm
Yagur et al., 2024	Retrospective cohort	Israel	32 endometriosis	NR	5.9	65	NR	NR	12 mo	0%	NR	Endometriosis subset of 12-month experience; no conversion
Mixed gynecology/learning curve												
Yap et al., 2024	Retrospective cohort	Panama	NR (144 mixed gyn)	NR	NR	NR	NR	NR	NR	0%	0%	Complex vs. non-complex patient comparison; EBL minimal and LOS short (qualitative)

Table 3 includes study entries across four procedure blocks

CD I-II = Clavien-Dindo grades I and II (minor complications); CD ≥ II = Clavien-Dindo grade ≥ II; Conv. = conversion to laparoscopy or laparotomy; CS = case series; CUSUM = cumulative sum analysis; EBL = estimated blood loss; EC = endometrial cancer; FU = follow-up duration (months unless otherwise stated); GI = gastrointestinal; gyn = gynecologic; hyst. = hysterectomy; IDEAL = Idea, Development, Exploration, Assessment, Long-term study framework; LC = learning curve; LOS = length of hospital stay; mo = months; NR = not reported in the source study; OR time = skin-to-skin operative time; POP = pelvic organ prolapse; PSM = propensity score matching; RAS = robot-assisted surgery; SCP = sacrocolpopexy. Values are median or mean as reported. Numeric fields report only directly extractable absolute values. Mixed-procedure cohorts are explicitly labelled; the same study may appear in more than one procedure block when separate extractable data were reported per procedure. ↑/↓ = directional finding not amenable to numeric extraction

Second, Da Vinci SP and HUGO possess the deepest gynecologic literatures. Senhance contributes the most informative per-case cost data. KANGDUO provides the only randomized trial among the newer platforms. Versius, hinotori, Dexter, SHURUI, Toumai, EDGE, Anovo, Maestro, and CARINA remain supported by much smaller and earlier-stage literatures. This asymmetry is consequential: a favorable signal for a platform with a mature program reflects a different level of evidence.

Third, the least mature domains across all platforms are economic evaluation, learning-curve quantification, and longer-term clinical follow-up. Even where cost or learning data exist, they are context-specific, procedure-specific, and derived from a small number of centers.

The present multi-platform synthesis complements and extends the existing single-platform and single-indication systematic reviews in this field. Prior meta-analyses have addressed robotic versus laparoscopic surgery for deep endometriosis [10] and the comparative effectiveness of single-site robotic and laparoscopic surgery across gynecologic indications [11]. Those analyses provided pooled effect estimates for narrow, well-defined comparisons but could not address the question this review asks: how does the emerging robotic landscape as a whole compare internally, and which domains of evidence are most critically underdeveloped? By synthesizing 92 studies across 13 platforms with a platform-organized structure, this review identifies asymmetries in evidentiary maturity that are invisible in single-platform or single-indication analyses.

### Platform category comparisons

Single-port systems share the goal of minimizing abdominal wall trauma and maximizing cosmetic outcomes but differ substantially in maturity. Da Vinci SP is the most mature, with evidence for short-term safety, operative learning, and oncologic feasibility, while SHURUI and EDGE remain in early institutional phases with favorable blood loss and cosmetic outcomes. The central unresolved question across this category is whether single-port access advantages translate into clinically meaningful patient benefit beyond the perioperative period - a question that Farinha et al. [97] framed in 2022 as asking whether emerging platforms were achieving the golden goals of improved outcomes, reduced costs, and broader access. At that time, evidence was even more sparse. The present review demonstrates that the evidence base has matured substantially, but the fundamental question of durable patient-level benefit remains unanswered for most systems.

**Table 4:** Senhance - surgical outcomes, cost data, and learning-curve information: per-study data

First author, year	Study design	Country	*N* (Senhance)	OR time (min)	Docking (min)	EBL (mL)	LOS (days)	FU	Conv. (%)	CD ≥ II (%)	Cost/LC notes
Hysterectomy/oncology											
Gueli Alletti et al., 2018	Prospective pilot study	Italy	10 (obese, EC)	110 (median)	10.5	100	2	NR	0%	0%	BMI 30–40; feasibility in obese; no conversion
Samalavicius et al., 2020	Prospective single-center cohort	Lithuania	30 gyn (100 total)	145 (mean, gyn)	NR	NR	4	NR	3.3%	6.7%	First 100-case mixed institutional experience; gyn subset
Abendstein et al., 2024	Retrospective 2-center cohort	Austria/Lithuania	295	95 (median)	3	NR	NR	NR	1%	2%	Multisite TLH; AE rate 2%; mature-phase OR time reflects post-adoption phase
Staib et al., 2025	Prospective registry (TRUST)	Multicenter (EU/USA)	609 gyn (3,239 total)	NR	NR	NR	NR	NR	4.5%	3.9%	Multi-specialty safety registry; gyn subset: 609 of 3,239 total cases; Conv. and CD = overall registry rates
Abendstein et al., 2025	Prospective registry subgroup (TRUST oncology cohort)	Multicenter (EU/USA)	966 (multi-disciplinary)	NR	NR	NR	NR	12	4.8%	NR	Oncology subgroup of TRUST registry; not gynecology-specific
Sacrocolpopexy											
Sassani et al., 2022	Case series	USA	25	210.2 (mean)	NR	35	NR	NR	0%	0%	LC signal: OR time 231.7→190.3 min first vs. second half (*p* < 0.05); 2 anatomical recurrences (asymptomatic)
Clark et al., 2023	Retrospective comparative cohort with PSM	USA	25	242.2 (mean)	NR	NR	NR	NR	0%	NR	da Vinci (multiport, *n* = 50); after adj.: Senhance cost ↓$908.33; capital costs excluded
Cost and learning curve studies											
Coussons et al., 2021	Retrospective cost analysis	USA	NR	NR	NR	NR	NR	NR	NR	NR	Instrument cost: $559 (Senhance) vs. $1,393 (da Vinci; *p* < 0.001); vs. LAVH $498; capital costs excluded
Hutchins et al., 2019	Prospective simulation study (FLS)	USA	16 participants	N/A	N/A	N/A	N/A	N/A	N/A	N/A	Simulation LC (peg transfer, precision cutting); residents, fellows, attendings all improved; haptic feedback: no detriment

Table 4 includes study entries across three blocks. Console time is not presented because it was reported in only 1 of 9 studies (Coussons 2021, a cost study rather than an outcome study; console time 91.5 min)

adj. = adjusted for OR time, blood loss, and concomitant procedures; AE = adverse event; BMI = body mass index; CD ≥ II = Clavien-Dindo grade ≥ II; Conv. = conversion to laparoscopy or laparotomy; EBL = estimated blood loss; EC = endometrial cancer; EU = Europe; FLS = Fundamentals of Laparoscopic Surgery simulation; FU = follow-up duration; gyn = gynecologic; IA = FIGO stage IA; LAVH = laparoscopic-assisted vaginal hysterectomy; LC = learning curve; LOS = length of hospital stay; N/A = not applicable (simulation study); NR = not reported in the source study; OR time = skin-to-skin operative time; PSM = propensity score matching; TLH = total laparoscopic hysterectomy; TRUST = prospective multicenter registry (Senhance/Asensus Surgical). Values are median or mean as reported. Numeric fields report only directly extractable absolute values. Console time omitted (reported in 1/9 studies; 89% NR)

Alternative multi-arm platforms represent a heterogeneous group of systems that differ in console configuration, arm deployment, and bedside workflow, but all aim to expand the technological landscape beyond conventional multiport robotic surgery. HUGO is the most extensively studied within this group, with sacrocolpopexy outcomes comparable to conventional laparoscopy [47] and endometriosis results broadly comparable to da Vinci in the first multicenter head-to-head comparison [51]. Versius has accumulated the broadest multinational validation within this group, including prospective IDEAL-staged evidence and registry-based data [64, 70]. Hinotori has demonstrated procedural safety and oncologic feasibility in Japan, but comparative studies have generally reported longer console and operative times than more established platforms, suggesting a potential gap that requires further study [71–77]. Dexter has so far been evaluated mainly in hysterectomy-focused series; its on-demand hybrid integration model may offer workflow flexibility in settings where a fully dedicated robotic pathway is not practical, although this potential advantage remains hypothetical and has not been formally tested [78–81]. The differing arm configurations, docking strategies, and bedside logistics of these systems also imply that implementation requires structured training not only for the console surgeon but for the entire operating-room team [5, 98]. Across this heterogeneous group, currently available comparative data support feasibility and acceptable short-term safety, but do not demonstrate clear superiority over established robotic or laparoscopic approaches.

Chinese-developed platforms reflect the rapid expansion of robotic innovation in China and may be particularly relevant in discussions of regional availability, and cost containment [82–92, 96]. Among these systems, KANGDUO stands out for providing the only randomized trial included in this review, showing non-inferiority to da Vinci Xi in endometrial cancer staging, together with an initial signal of lower hospitalization and surgical costs in retrospective comparative analysis [89, 90]. For the remaining Chinese-developed platforms, the evidence is still largely limited to feasibility studies, pilot series, or early clinical reports [82–88, 91, 92, 96]. Any economic interpretation of these data should remain cautious, as the reported cost data arise from healthcare systems and reimbursement structures that may not be generalizable to European or North American settings [89, 90].

Finally, platforms such as Anovo and Maestro are designed for specific access strategies, addressing narrower technical concepts rather than broad multi-indication robotic adoption. Anovo was specifically designed for robotic vaginal natural orifice surgery and has shown that robotic transvaginal hysterectomy and bilateral salpingo-oophorectomy are feasible, with very low blood loss and limited postoperative pain in early prospective studies [93, 95]. Maestro has generated only minimal gynecologic evidence: two sacrocolpopexy procedures were included in a multispecialty IDEAL 2a solo-surgery cohort, but procedure-specific gynecologic outcomes were not separately reported. Nevertheless, its inclusion is conceptually relevant because Maestro represents an AI-enabled surgical assistant capable of physical interaction with the operative field, highlighting the potential future role of digitally assisted and progressively autonomous surgical workflows [95]. Toumai currently provides proof of feasibility for telesurgery in gynecology, including a completed European telesurgical hysterectomy, but the available evidence remains extremely limited [85–87]. The potential role of telesurgery, remote collaboration, and AI-assisted surgical guidance may ultimately intersect with several robotic platforms rather than remaining confined to one dedicated system [99]. At present, however, whether these enabling technologies translate into clinically meaningful benefit sufficient to justify their additional complexity and cost remains uncertain and will require larger, targeted studies [85–87, 93–95, 99].

A further implication of platform diversification concerns surgical access strategy: emerging systems may reshape abdominal entry and trocar placement beyond conventional multiport layouts. Single-port systems concentrate entry through an umbilical or transvaginal route, whereas modular multi-arm platforms require procedure-specific planning of trocar spacing, arm configuration, assistant access, and collision avoidance [5, 6, 15–17, 69]. These architectural differences may affect setup efficiency, ergonomics, and access-related adverse events; yet current studies rarely report access-specific metrics. Future evaluations should therefore systematically document access route, trocar configuration, docking strategy, assistant-port use, arm collisions, and need for additional ports. This is especially relevant during the learning-curve phase, when suboptimal access planning - rather than intrinsic platform limitations - may compromise operative workflow and surgical safety [5, 6, 52–55].

**Table 5:** Other emerging platforms (Versius, hinotori, Dexter, SHURUI, Toumai, KANGDUO, EDGE SP1000, Anovo/Hominis, Maestro, CARINA) - per-study surgical outcomes with integrated cost and learning-curve data

First author, year	Country	Study design	Procedure	*N*	OR time (min)	EBL (mL)	LOS (days)	FU	Conv. (%)	CD ≥ II (%)	Key findings/cost/LC
Versius											
Puntambekar et al., 2021	India	Prospective case series	Radical hyst. (cervical/EC)	30	104 (mean)	60	2.1	NR	0%	6.7% (UVF)	LN yield 24.7; first Versius RRH; 2 ureterovaginal fistulas treated postoperatively
Borse et al., 2022	India	Prospective cohort (IDEAL 2b)	Total laparoscopic hyst.	144	NR	NR	NR	NR	1.4%	NR	3 hospitals; AEs < 5%; 1 device-related SAE; no open conversion
Sadlecki et al., 2025	Poland	Retrospective comparative cohort	Hyst. (benign + malignant)	21	134.6 (mean)	NR	NR	NR	0%	NR	laparoscopy comparator; OR time longer vs. laparoscopy (*p* < 0.01); other outcomes comparable
Uccella et al., 2025	Italy	Prospective case series	EC staging + SLN biopsy	14	122 (median)	NR	NR	NR	0%	0%	SLN detection 100%; first Versius EC staging; no readmissions/reoperations
Panico et al., 2025	Italy	Prospective single-center cohort	Sacrocolpopexy	20	174 (median)	NR	NR	3 mo	0%	0%	Nerve-sparing; 90% complete anatomical correction at 3 months; PROs improved
Sighinolfi et al., 2024	Italy	Retrospective multicenter cohort	Pelvic surgery (mixed)	11	NR	NR	NR	NR	0%	NR	11 gyn (42 pelvic total); 9 adnexectomies, 2 prolapse surgeries; no conversions; 2 system alarms/malfunctions
Soumpasis et al., 2023	UK/Multicenter	Prospective registry	Hyst. (registry subset)	324	NR	NR	NR	90-day	NR	NR	2,083 total cases in Versius Surgical Registry (324 gyn); reported low SAE, conversion, and 90-day mortality overall
Hinotori											
Togami et al., 2023	Japan	Case series	Total hyst. (mixed indications)	12	214 (median)	22	6	NR	0%	NR	Indications: EC, fibroids, atypical hyperplasia, adenosarcoma, CIN; 1 postoperative pelvic infection
Matsuura et al., 2024	Japan	Retrospective comparative study, 3-system	Total hyst.	10	123 (mean)	NR	NR	NR	0%	0%	da Vinci SP and HUGO comparators; OR time longer vs. SP/HUGO (*p* = 0.031)
Togami et al., 2025	Japan	Retrospective comparative cohort	Hyst. + SLN biopsy for EC	20	NR	NR	NR	NR	0%	0%	da Vinci Xi comparator (*n* = 214); comparable outcomes; pre-console interval longer (*p* = 0.004)
Togami et al., 2025	Japan	Retrospective comparative cohort	Sacrocolpopexy	22	286 (mean)	NR	NR	NR	0%	NR	da Vinci Xi comparator (221 min; *p* < 0.001); OR time longer; functional outcomes comparable
Ichino et al., 2024	Japan	Retrospective comparative cohort	Sacrocolpopexy	15	266 (mean)	NR	NR	NR	0%	NR	da Vinci comparator (229 min); longer OR + console time; comparable OABSS and recurrence
Fukumoto et al., 2025	Japan	Retrospective single-surgeon	Sacrocolpopexy	30	148 (mean)	NR	NR	7 mo	0%	NR	da Vinci comparator; suture time 76 vs. 60 s/stitch (*p* < 0.005); may explain OR time gap
Togami et al., 2024	Japan	Retrospective case series	Mixed gyn (hyst/SCP/EC)	69	NR	NR	NR	NR	0%	NR	da Vinci Xi comparator (*n* = 332); console time 173 min hinotori vs. 156 min da Vinci Xi (*p* = 0.047); no difference in total OR time, EBL, or LOS; 401 total: 43 benign, 88 POP, 270 low-risk EC
Nagata et al., 2025	Japan	Retrospective comparative study, 4-system	Total hysterectomy	NR	NR	NR	NR	NR	0%	NR	da Vinci X or Xi and HUGO comparators; no significant overall cohort difference
Dexter											
Alkatout et al., 2024	Germany	Case report	Subtotal hyst. + cervico-sacrocolpopexy (bikini line)	1	150	10	2	NR	0%	0%	First below-bikini-line RAH; docking 6 min
Gulz et al., 2025	Switzerland	Prospective multicenter single-arm study	Hyst. + adnexal surgery	52	121.9 (mean)	87.8	2	6w	0%	1.9% (CD IIIb)	Docking 3.8 min; no device-related AEs
Imboden et al., 2025	Switzerland	Prospective multicenter study (IDEAL)	Hyst. (benign + low-risk malignant)	34	125.5 (median)	100	2	1 m	8.8%	5.9% (CD IIIb)	No device-related AEs; 3 cases completed laparoscopically by surgeon choice
Imboden et al., 2025	Switzerland	Retrospective case series	Hyst. (EC, endometriosis, fibroids)	24	171.9 (mean)	130.8	4	NR	0%	16.7% (CD III)	On-demand hybrid laparoscopic-robotic workflow; no device-related AEs
SHURUI/SR-ENS-600											
Hu et al., 2024	China	Prospective case series (IDEAL 2a)	Single-site gyn surgery (mixed)	10	NR	NR	NR	3 mo	0%	0%	First-in-human; no serious complications at 3 months
Chang et al., 2024	China	Prospective multicenter trial	Single-site gyn surgery (mixed)	63	157.0 (mean)	63.9	3.6	NR	0%	0%	6 centers; ovarian cysts, myomas, CIN, EC; good cosmesis; no conversions/readmissions
Zhao et al., 2025	China	Retrospective cohort	Single-site hysterectomy	19	NR	40.5	NR	NR	0%	0%	da Vinci Xi (single-site) and da Vinci Xi (multiport) comparators; EBL ↓ vs. both (*p* < 0.05); time to flatus ↓
Toumai											
Pazzaglia et al., 2025	Belgium	Case report (telesurgery)	Total hyst. + bilateral salpingectomy	1	74	NR	2	18 days	0%	0%	First European telesurgical gynecologic procedure; network latency 20 ms
Pasquini et al., 2026	Europe	Pilot study	Total hysterectomy	1	NR	NR	NR	NR	NR	NR	Pilot European evaluation; full outcome data not reported in publication
Sighinolfi et al., 2025	China	Retrospective multispecialty	Mixed (telesurgery)	NR gyn	NR	NR	NR	NR	NR	NR	Multi-specialty series (*n* = 66 total); gyn network delay ≈ 61 ms; gyn subset not separately reported
KANGDUO/KD-SR-01											
Pi et al., 2025	China	Retrospective comparative cohort	Mixed gyn (hyst, ooph, EC, cervical)	242	NR	NR	6	NR	0%	0%	Laparoscopy comparator (*n* = 164); OR time, EBL, hospitalization, and drainage all ↓ vs. laparoscopy; no CD ≥ III; breakdown: 144 hyst, 25 ooph (benign/borderline), 25 ooph (bilateral), 24 EC staging, 24 cervical cancer
Li et al., 2025	China	Randomized non-inferiority trial	EC staging	50 KD/49 DV	NR	NR	NR	NR	0%	NR	Non-inferior to da Vinci Xi for LN yield (primary endpoint); docking slightly longer (5.39 vs. 4.34 min)
Liu et al., 2025	China	Retrospective comparative cohort	EC surgery (staging)	125 KD/86 DV	111.1 (KD)/95.8 (DV)	NR	5.0 (KD)/4.4 (DV)	NR	0%	NR	da Vinci Xi comparator; OR time longer with KD (111.1 vs. 95.8 min; *p* = 0.001); LOS longer with KD (5.0 vs. 4.4 days; *p* = 0.001); console KD 70.3 vs. DV 56.6 min; EBL and CD I-II: no significant difference; total costs lower in KD group (*p* < 0.05)
EDGE SP1000											
Gong et al., 2024	China	Pilot case series	Single-port gyn surgery (benign + malignant)	33	105.5 (mean)	34.6	NR	NR	0%	NR	1 postoperative fever; satisfactory wound healing and cosmesis
Chen et al., 2024	China	Prospective single-arm trial	Single-port gyn surgery (mixed)	18	190.1 benign/254.4 malig.	25	NR	1 mo	0%	0%	8 malignant cases; no assistant ports; high cosmetic satisfaction at 1 month
ANOVO/HOMINIS											
Lowenstein et al., 2020	Israel	Prospective 2-center study	RvNOTES BSO	8	45 (median)	10	NR	NR	0%	0%	Surgeon usability 5/5; VAS pain 1/10 at 24 h; OR time decreasing over series
Lowenstein et al., 2021	Israel	Prospective 2-center study	RvNOTES total hysterectomy	30	57 (median)	50	3	6 wk	0%	0%	No intraoperative complications; VAS pain 3/10 at 24 h; complete vaginal cuff healing at 6 weeks
Maestro											
Mercoli et al., 2026	France	Prospective multicenter study (IDEAL 2a)	Mixed; sacrocolpopexy included	45 (2 gyn)	43.6 (mean)	NR	< 1	NR	2.2%	0%	AI-powered solo surgery; setup 6.1 min; no device-related AEs; overall data, no SCP-specific outcomes extractable
carina											
Liu et al., 2025	China	Retrospective case series	Radical hyst. (cerv.) + hyst. (EC)	16	211.9 cerv./153.3 EC	30 cerv./20 EC	NR	NR	0%	0%	Docking 5.75 min; console time 154.6 (cerv.)/98.7 (EC) min; no device-related complications

Table 5 includes study entries across 10 platforms. Geographic origin is shown to support assessment of generalizability across regulatory contexts

AE = adverse event; AI = artificial intelligence; BSO = bilateral salpingo-oophorectomy; CD I-II = Clavien-Dindo grades I and II; CD III = Clavien-Dindo grade III; CD IIIb = Clavien-Dindo grade IIIb; CD ≥ II = Clavien-Dindo grade ≥ II; cerv. = cervical cancer group; CIN = cervical intraepithelial neoplasia; Conv. = conversion to laparoscopy or laparotomy; CS = case series; DV = da Vinci; EBL = estimated blood loss; EC = endometrial cancer; FU = follow-up duration (months unless stated); gyn = gynecologic; hyst. = hysterectomy; IDEAL = Idea, Development, Exploration, Assessment, Long-term study framework; KD = KangDuo; LC = learning curve; LN = lymph node; LOS = length of hospital stay; malig. = malignant; mo = months; NR = not reported in the source study; OABSS = Overactive Bladder Symptom Score; ooph. = oophorectomy; OR time = skin-to-skin operative time; POP = pelvic organ prolapse; PROs = patient-reported outcomes; RAH = robot-assisted hysterectomy; RAS = robot-assisted surgery; RCT = randomized controlled trial; RRH = robot-assisted radical hysterectomy; RvNOTES = robotic vaginal natural orifice transluminal endoscopic surgery; SAE = serious adverse event; SCP = sacrocolpopexy; SLN = sentinel lymph node; SP = single-port; UVF = ureterovaginal fistula; VAS = visual analog scale; wk = weeks. Values are median or mean as reported. Numeric fields report only directly extractable absolute values. Cross-platform comparative studies are listed under each relevant platform. Docking and console times are reported in the “Key findings” column when available because reporting was heterogeneous across platforms. ↑/↓ = directional finding not amenable to numeric extraction

### Learning-curve evidence: synthesis and implications

The most robust learning-curve evidence comes from da Vinci SP and HUGO. For da Vinci SP, current data suggest proficiency in hysterectomy after approximately 8–13 cases for surgeons with prior robotic experience, and after approximately 50 cases for those transitioning from conventional multiport surgery [21, 40]. For HUGO, docking proficiency appears achievable after approximately 15–20 cases, while the console surgeon’s operative-time plateau is reached earlier - by case 5 for experienced operators and approximately 15 for robotics-naive surgeons - indicating that setup and surgical execution curves proceed at different rates [52, 54, 55]. For Senhance and hinotori, indirect learning-curve data suggest a manageable adoption phase, but without CUSUM analyses these data are less methodologically robust. For SHURUI, Dexter, EDGE, Anovo, Toumai, KANGDUO, Versius, CARINA, and Maestro, formal gynecologic learning-curve data are essentially absent, representing a critical evidence gap for institutions considering adoption.

Three additional methodological points deserve emphasis. First, at least three distinct learning curves should be conceptually recognized: (i) console surgeon proficiency with instrument control and procedure-specific technique, (ii) bedside assistant and team workflow adaptation, and (iii) institutional docking, setup, and turnover efficiency [52]. These components likely stabilize at different rates and should not be compressed into a single estimate of “platform learning curve.” Available data partially support this disaggregation. Beyond the HUGO findings noted above, da Vinci SP series showed consistently short docking times (median 5 min) even in early adoption [15], whereas overall surgical proficiency required 8–13 cases [21, 40], suggesting rapid setup mastery relative to console execution. For hinotori, the dissociation was demonstrated directly: the pre-console interval was significantly longer than with da Vinci Xi, whereas console time was comparable [72], confirming that setup efficiency lagged behind surgical execution during early adoption. No included study, however, explicitly isolates team-level or bedside assistant adaptation as an independent outcome; future learning-curve analyses should attempt to disaggregate this third dimension, as composite metrics risk masking clinically relevant differences among workflow components.

Second, simulation-based training may compress the early phases of these curves: structured laboratory experience with Senhance haptic feedback accelerated skill acquisition and improved performance metrics in trainee populations across all experience levels [63], suggesting that pre-clinical training protocols should be systematically incorporated into adoption programs for all platforms. Third, the comprehensive review by Komatsu et al. [6] underlines that parallel platform proliferation places particular strain on institutional training infrastructure, a concern that is likely to intensify as additional systems enter gynecologic practice.

### Economic evidence: synthesis and limitations

Economic evidence is concentrated in Senhance and KANGDUO and largely absent for all other platforms. The Senhance cost data demonstrate a reproducible instrument-cost advantage relative to da Vinci in both hysterectomy and sacrocolpopexy [61, 62]. However, an instrument-cost advantage should not be equated with full economic superiority unless capital acquisition, maintenance, depreciation, staff training, operating-room time, turnover efficiency, conversion costs, and downstream clinical outcomes are included in the analysis. The demonstrated per-case advantage may therefore be smaller or reversed in a comprehensive evaluation. The KANGDUO cost findings are directionally consistent but reflect the Chinese healthcare cost structure and may not transfer to other systems. For HUGO, Versius, hinotori, Dexter, da Vinci SP, and all remaining platforms, gynecology-specific economic evidence is essentially missing. A comprehensive economic review of next-generation robotic surgical systems [100] confirmed this structural gap: no peer-reviewed full health technology assessment incorporating all of the above cost domains has been published for any emerging gynecologic robotic platform. A further limitation is the absence of a common cost basis across national healthcare systems. The Senhance cost advantage data derive predominantly from US and European centers, the KANGDUO cost findings reflect the Chinese public-hospital pricing structure, and no cross-national cost comparison has been performed. Therefore, direct comparison of absolute costs is methodologically unsound, given differences in capital acquisition, instrument pricing, labor and operating-room costs, insurance and reimbursement models, and currency fluctuations. Cost advantages observed within one regulatory and economic context may not be transferable to another without system-specific modeling. Accordingly, until full health-technology assessments incorporating all relevant cost domains are conducted in specific healthcare settings, the economic findings of this review should be interpreted as directional signals rather than transferable evidence.

### Long-term clinical outcomes: a critical evidence gap

The absence of long-term clinical outcome data is a key limitation that deserves separate emphasis. For hysterectomy, short-term perioperative endpoints may be adequate to establish early feasibility, although durable functional and quality-of-life data remain desirable. For sacrocolpopexy, myomectomy, endometriosis surgery, and oncologic staging, however, meaningful clinical evaluation requires data on prolapse recurrence, fertility, pain control, quality of life, and oncologic follow-up - none of which are systematically reported for any emerging platform. Until such evidence becomes available, current conclusions remain confined to short-term technical and safety outcomes.

### Interpretation in context

Cross-platform interpretation requires methodological caution because included studies are not directly commensurate. Unlike drug evaluation, surgical robotic platform development and clinical integration are iterative, context-dependent processes influenced by device evolution, surgeon learning curves, team workflow, human factors, and institutional implementation conditions [14]. Apparent differences in operative time, blood loss, or docking time across platforms may therefore reflect case mix, adoption phase, surgeon experience, and local workflow rather than intrinsic platform characteristics. In some reports, newer systems were introduced by teams with extensive prior robotic experience, potentially compressing the observed learning curve; in others, platforms were evaluated during early local implementation, when setup inefficiency may have prolonged operative times. For this reason, cross-study ranking of platforms based on aggregate operative metrics would be methodologically inappropriate and was explicitly avoided in this synthesis.

Hysterectomy and sacrocolpopexy dominate the literature, whereas myomectomy, endometriosis surgery, and oncologic staging are represented by smaller and less standardized subgroups. Long-term prolapse recurrence, fertility outcomes after myomectomy, durable pain control after endometriosis surgery, and oncologic endpoints beyond immediate perioperative feasibility are rarely reported. The concentration of evidence in expert academic centers further limits generalizability: implementation in lower-volume centers or healthcare systems with different resource constraints may produce different efficiency, safety, and cost profiles, as illustrated by the HUGO sacrocolpopexy series from a novice robotic team [48].

Publication bias is a further interpretive challenge. Institutions adopting new systems tend to be enthusiastic early adopters with existing robotic expertise and are more likely to report favorable results, while unfavorable early experiences, abandoned programs, and non-adopting centers are systematically underrepresented in the published literature. This structural bias means that the feasibility and safety profile of each platform may not provide a population-representative estimate. The transition from single-center reports to multi-center registry data - as exemplified by the Versius Surgical Registry [70] and the Senhance TRUST registry [59] - represents a necessary maturation step that most platforms in this review have not yet completed.

### Strengths and limitations

Strengths of this review include a focused and clinically relevant question; synthesis of evidence from 92 studies across 13 architecturally diverse platforms; organization by platform rather than a generic robotic surgery category; duplicate study selection, data extraction, and design-sensitive risk-of-bias assessment; a conservative extraction strategy in which non-reported variables were not inferred; and inclusion of platforms across multiple regulatory jurisdictions, reflecting the global nature of the emerging robotic field.

Several limitations should be recognized. The evidence base was dominated by retrospective single-center studies with modest sample sizes, short follow-up, limited use of concurrent control groups, and incomplete adjustment for confounding. These methodological characteristics collectively constrain confidence in the reported findings: the predominantly moderate risk-of-bias profile means that most favorable perioperative signals should be regarded as hypothesis-generating rather than confirmatory. Short and incompletely reported follow-up is a further systematic weakness: for oncologic indications, long-term survival and recurrence data are conspicuously absent, and for reconstructive procedures, anatomical and functional outcomes beyond 6–12 months are largely unavailable, preventing any meaningful assessment of durability. Platform-specific case mix was heterogeneous even within the same procedure category, and outcome definitions were inconsistent across studies, particularly for operative time components, recovery measures, complication reporting, and economic endpoints. The lack of standardized reporting conventions in robotic surgery limited direct comparison across platforms and procedures. Finally, the inclusion of multiple observational comparative studies carries an inherent risk of residual confounding related to institutional experience, adoption phase, surgeon expertise, and case-mix differences that propensity-score methods can reduce but not eliminate.

### Clinical and research implications

A central translational recommendation of this review is the establishment of a prospective multi-platform gynecologic robotic surgery registry. The current fragmentation of evidence across platforms, study designs, and healthcare systems cannot be resolved by single-center studies alone. Such an initiative - designed to collect standardized data across all commercially available systems simultaneously, analogous to what the Versius registry [70] has achieved for a single platform but extending across competing systems with a common data dictionary - would represent a transformative step for the field, enabling genuine comparative-effectiveness analyses on real-world populations rather than expert-center cohorts.

For clinical practice, the most defensible conclusion is that several emerging platforms can be adopted safely for selected gynecologic procedures in appropriately experienced centers, but platform selection should remain indication-specific and institution-specific rather than brand-driven. Practical decisions should account for the center’s current case mix and volume, available expertise, docking workflow, the anticipated balance of reusable and disposable instrumentation, and whether the anticipated benefit is procedure-specific rather than assumed to apply universally. Platforms should not be adopted on the basis of marketing claims alone, and centers without prior robotic experience should exercise particular caution in interpreting literature generated at high-volume expert centers.

For future research, the field should move beyond proof-of-feasibility studies toward comparative, standardized, and longitudinal evidence. Studies should adopt validated perioperative outcome definitions, explicit Clavien-Dindo complication grading, predefined conversion criteria, and procedure-specific follow-up of sufficient duration to capture recurrence, functional outcomes, quality of life, and oncologic endpoints when applicable. Economic evaluations should use transparent methods and separately report capital acquisition, maintenance, instrument costs, operating-room time, hospital stay, and downstream resource use. Prospective learning-curve studies should prespecify surgeon experience, define proficiency using objective criteria, and report outcomes across multiple surgeons and centers. The IDEAL and IDEAL-D frameworks provide a useful structure for the orderly generation and reporting of surgical and device innovation evidence, from early development to comparative evaluation and long-term monitoring [12–14].

Future studies should also establish consensus on a minimum outcome reporting set for robotic gynecologic surgery and investigate how emerging technologies including AI-assisted image guidance and telesurgery will intersect with the evolving robotic platform landscape [99].

## Conclusions

Emerging robotic platforms have materially broadened the technical landscape of minimally invasive gynecologic surgery, offering architecturally diverse solutions for single-port access, modular multi-arm surgery, transvaginal procedures, and teleoperation. The current evidence, however, remains uneven, predominantly observational, and largely limited to short-term perioperative outcomes from expert centers. Da Vinci SP and HUGO hold the most substantial gynecologic literatures; Senhance contributes the clearest gynecology-specific per-case cost data; KANGDUO provides the only randomized trial among the newer platforms and initial economic advantage data within its regulatory context. For most other systems, the literature sits at the level of feasibility and early implementation. The present review supports cautious, platform-specific adoption in experienced centers, guided by procedure-appropriate evidence rather than platform brand, and underscores the urgent need for better comparative, economic, and longitudinal evidence before any broad claim of superiority or generalized value can be justified.

## Supplementary Information

Below is the link to the electronic supplementary material.

Supplementary Material 1

Supplementary Material 2

## Data Availability

All data supporting the findings of this study are available within the paper and its Supplementary Information. Further enquiries can be directed to the corresponding author.
