# Supplementary material for: Emerging robotic platforms in gynecologic surgery: a systematic review
Source: J Robot Surg. 2026 Jun 22;20(1):634. doi: 10.1007/s11701-026-03590-4 (PMC13284037; doi:10.1007/s11701-026-03590-4)
Supplement: Supplementary file 1 — Supplementary Material 1 [file 11701_2026_3590_MOESM1_ESM.docx]

**Supplementary Appendix S1 - Full Search Strategies**

Searches were conducted simultaneously on 4 April 2026 in PubMed, Scopus, and Web of Science. The same conceptual structure was applied across all three databases: (1) a platform block listing all emerging robotic systems by trade name, manufacturer name, or model identifier; (2) a gynecologic procedure block covering relevant anatomical terms and procedure names; and (3) a robotic surgery methodology block. Blocks were combined with Boolean AND. Database-specific syntax was applied as required by each platform. No date restrictions were applied. The PubMed search additionally used the "humans" MeSH term to limit results to human studies; equivalent document-type or subject-area filters were applied in Scopus and Web of Science at the database interface level. All three searches were independently re-verified by both reviewers before the de-duplication step.

## S1.1 PubMed Search String

Database: MEDLINE via PubMed (https://pubmed.ncbi.nlm.nih.gov/)

Search field: [tiab] = title and abstract

Records retrieved: 224

( "Hugo"[tiab] OR "Hugo RAS"[tiab] OR "Medtronic robotic"[tiab] OR "Versius"[tiab] OR
"CMR Surgical"[tiab] OR "Hinotori"[tiab] OR "hinotori surgical robot"[tiab] OR
"Senhance"[tiab] OR "Asensus Surgical"[tiab] OR "Dexter"[tiab] OR "Distalmotion"[tiab] OR
"REVO-I"[tiab] OR "Revo-i robotic"[tiab] OR "Meere Company"[tiab] OR
"Meerecompany"[tiab] OR "Avatera"[tiab] OR "avateramedical"[tiab] OR "Carina"[tiab] OR
"Surgica Robotics"[tiab] OR "Ronovo"[tiab] OR "KangDuo"[tiab] OR "Kangduo SR"[tiab] OR
"KangDuo SR1000"[tiab] OR "KangDuo SR2000"[tiab] OR "EDGE MP1000"[tiab] OR
"Cornerstone Robotics"[tiab] OR "Toumai"[tiab] OR "Toumai robotic"[tiab] OR
"MicroPort MedBot"[tiab] OR "Microhand"[tiab] OR "Micro Hand S"[tiab] OR
"SSi Mantra"[tiab] OR "SSI Mantra"[tiab] OR "SS Innovations"[tiab] OR "Anovo"[tiab] OR
"Hominis"[tiab] OR "Memic"[tiab] OR "Momentis Surgical"[tiab] OR "Surgenius"[tiab] OR
"SHURUI"[tiab] OR "SR-ENS-600"[tiab] OR "Ottava"[tiab] OR "Ethicon robotic"[tiab] OR
"da Vinci SP"[tiab] OR "davinci SP"[tiab] OR "single-port robotic"[tiab] OR
"single port robotic"[tiab] OR "da Vinci 5"[tiab] OR "davinci 5"[tiab] OR
"da vinci five"[tiab] OR "DV5"[tiab] )
AND
( "gynecolog*"[tiab] OR "gynaecolog*"[tiab] OR "hysterectomy"[tiab] OR
"hysterectomies"[tiab] OR "myomectomy"[tiab] OR "myomectomies"[tiab] OR
"endometri*"[tiab] OR "uterus"[tiab] OR "uterine"[tiab] OR "cervix"[tiab] OR
"cervical"[tiab] OR "ovary"[tiab] OR "ovarian"[tiab] OR "adnex*"[tiab] OR
"salpingo*"[tiab] OR "salpingectomy"[tiab] OR "oophorectomy"[tiab] OR
"fallopian tube"[tiab] OR "vulva*"[tiab] OR "vagina*"[tiab] OR
"parametrectomy"[tiab] OR "trachelectomy"[tiab] OR "radical hysterectomy"[tiab] OR
"pelvic lymph node"[tiab] OR "pelvic lymphadenectomy"[tiab] OR
"paraaortic lymphadenectomy"[tiab] OR "para-aortic lymphadenectomy"[tiab] OR
"sentinel lymph node"[tiab] OR "sacrocolpopexy"[tiab] OR "sacrocolpospexy"[tiab] OR
"pelvic organ prolapse"[tiab] OR "endometriosis"[tiab] OR "uterine fibroid*"[tiab] OR
"uterine leiomyoma*"[tiab] OR "cytoreductive surgery"[tiab] OR "debulking"[tiab] OR
"surgical staging"[tiab] OR "staging surgery"[tiab] )
AND
( "robotic"[tiab] OR "robot-assisted"[tiab] OR "robotic-assisted"[tiab] OR
"robotic surgery"[tiab] OR "robotic system"[tiab] OR "robotic platform"[tiab] )
AND "humans"[MeSH Terms]

## S1.2 Scopus Search String

Database: Scopus (https://www.scopus.com/)

Search field: TITLE-ABS-KEY = searches title, abstract, and author keywords

Records retrieved: 320

TITLE-ABS-KEY (
 ( "Hugo" OR "Hugo RAS" OR "Medtronic robotic" OR "Versius" OR "CMR Surgical" OR
 "Hinotori" OR "hinotori surgical robot" OR "Senhance" OR "Asensus Surgical" OR
 "Dexter" OR "Distalmotion" OR "REVO-I" OR "Revo-i robotic" OR "Meere Company" OR
 "Meerecompany" OR "Avatera" OR "avateramedical" OR "Carina" OR "Surgica Robotics" OR
 "Ronovo" OR "KangDuo" OR "Kangduo SR" OR "KangDuo SR1000" OR "KangDuo SR2000" OR
 "EDGE MP1000" OR "Cornerstone Robotics" OR "Toumai" OR "Toumai robotic" OR
 "MicroPort MedBot" OR "Microhand" OR "Micro Hand S" OR "SSi Mantra" OR
 "SSI Mantra" OR "SS Innovations" OR "Anovo" OR "Hominis" OR "Memic" OR
 "Momentis Surgical" OR "Surgenius" OR "SHURUI" OR "SR-ENS-600" OR "Ottava" OR
 "Ethicon robotic" OR "da Vinci SP" OR "davinci SP" OR "single-port robotic" OR
 "single port robotic" OR "da Vinci 5" OR "davinci 5" OR "da vinci five" OR "DV5" )
 AND
 ( "gynecolog*" OR "gynaecolog*" OR "hysterectomy" OR "hysterectomies" OR
 "myomectomy" OR "myomectomies" OR "endometri*" OR "uterus" OR "uterine" OR
 "cervix" OR "cervical" OR "ovary" OR "ovarian" OR "adnex*" OR "salpingo*" OR
 "salpingectomy" OR "oophorectomy" OR "fallopian tube" OR "vulva*" OR "vagina*" OR
 "parametrectomy" OR "trachelectomy" OR "radical hysterectomy" OR
 "pelvic lymph node" OR "pelvic lymphadenectomy" OR "paraaortic lymphadenectomy" OR
 "para-aortic lymphadenectomy" OR "sentinel lymph node" OR "sacrocolpopexy" OR
 "sacrocolpospexy" OR "pelvic organ prolapse" OR "endometriosis" OR
 "uterine fibroid*" OR "uterine leiomyoma*" OR "cytoreductive surgery" OR
 "debulking" OR "surgical staging" OR "staging surgery" )
 AND
 ( "robotic" OR "robot-assisted" OR "robotic-assisted" OR "robotic surgery" OR
 "robotic system" OR "robotic platform" )
)

## S1.3 Web of Science Search String

Database: Web of Science Core Collection (https://www.webofscience.com/)

Search field: TS = Topic (searches title, abstract, author keywords, and Keywords Plus®)

Records retrieved: 349

TS=( "Hugo" OR "Hugo RAS" OR "Medtronic robotic" OR "Versius" OR "CMR Surgical" OR
 "Hinotori" OR "hinotori surgical robot" OR "Senhance" OR "Asensus Surgical" OR
 "Dexter" OR "Distalmotion" OR "REVO-I" OR "Revo-i robotic" OR "Meere Company" OR
 "Meerecompany" OR "Avatera" OR "avateramedical" OR "Carina" OR "Surgica Robotics" OR
 "Ronovo" OR "KangDuo" OR "Kangduo SR" OR "KangDuo SR1000" OR "KangDuo SR2000" OR
 "EDGE MP1000" OR "Cornerstone Robotics" OR "Toumai" OR "Toumai robotic" OR
 "MicroPort MedBot" OR "Microhand" OR "Micro Hand S" OR "SSi Mantra" OR
 "SSI Mantra" OR "SS Innovations" OR "Anovo" OR "Hominis" OR "Memic" OR
 "Momentis Surgical" OR "Surgenius" OR "SHURUI" OR "SR-ENS-600" OR "Ottava" OR
 "Ethicon robotic" OR "da Vinci SP" OR "davinci SP" OR "single-port robotic" OR
 "single port robotic" OR "da Vinci 5" OR "davinci 5" OR "da vinci five" OR "DV5" )
AND
TS=( "gynecolog*" OR "gynaecolog*" OR "hysterectomy" OR "hysterectomies" OR
 "myomectomy" OR "myomectomies" OR "endometri*" OR "uterus" OR "uterine" OR
 "cervix" OR "cervical" OR "ovary" OR "ovarian" OR "adnex*" OR "salpingo*" OR
 "salpingectomy" OR "oophorectomy" OR "fallopian tube" OR "vulva*" OR "vagina*" OR
 "parametrectomy" OR "trachelectomy" OR "radical hysterectomy" OR
 "pelvic lymph node" OR "pelvic lymphadenectomy" OR "paraaortic lymphadenectomy" OR
 "para-aortic lymphadenectomy" OR "sentinel lymph node" OR "sacrocolpopexy" OR
 "sacrocolpospexy" OR "pelvic organ prolapse" OR "endometriosis" OR
 "uterine fibroid*" OR "uterine leiomyoma*" OR "cytoreductive surgery" OR
 "debulking" OR "surgical staging" OR "staging surgery" )
AND
TS=( "robotic" OR "robot-assisted" OR "robotic-assisted" OR "robotic surgery" OR
 "robotic system" OR "robotic platform" )

## S1.4 Search Summary

| Database | Search date | Records retrieved |
| --- | --- | --- |
| PubMed (MEDLINE) | 4 April 2026 | 224 |
| Scopus | 4 April 2026 | 320 |
| Web of Science | 4 April 2026 | 349 |
| Total (before de-duplication) |  | 893 |
| After de-duplication |  | **408** |
