# Supplementary material for: Emerging robotic platforms in gynecologic surgery: a systematic review
Source: J Robot Surg. 2026 Jun 22;20(1):634. doi: 10.1007/s11701-026-03590-4 (PMC13284037; doi:10.1007/s11701-026-03590-4)
Supplement: Supplementary file 2 — Supplementary Material 2 [file 11701_2026_3590_MOESM2_ESM.docx]

**Supplementary Appendix S2 – Risk of Bias Assessment**

This appendix reports the study-level risk-of-bias and critical-appraisal assessment for all studies included in the systematic review. All assessments were conducted independently by two reviewers; disagreements were resolved by discussion and consensus. Design-appropriate instruments were selected for each study: the Revised Cochrane Risk of Bias tool (RoB 2) for the single randomized trial; the JBI Cohort Checklist for non-randomized comparative cohort studies; the JBI Case Series Checklist for single-arm clinical series, feasibility studies, and registries; the JBI Case Report Checklist for single-patient reports; and the JBI Analytical Cross-Sectional Checklist for the one simulation-based learning-curve study.

Because the JBI tools are critical-appraisal instruments rather than rule-based risk-of-bias classifiers, the overall concern level was assigned conservatively. Item-level responses informed, but did not mechanically determine, the overall judgment, which also considered intrinsic design limitations, sample size, absence of comparator, early feasibility status, and generalizability of the evidence. Studies with clearly matched or adjusted comparative designs were rated low concern. Most retrospective uncontrolled or unadjusted designs were rated moderate concern. Studies with major comparability problems, a narrow case mix combined with substantially underreported methods, or important biases in outcome assessment were rated high concern. Single case reports were conservatively retained in the moderate-concern category even when reporting was detailed, reflecting their intrinsic design limitations.

## Appraisal tools used

| **Tool** | **Applied to** | **n studies** |
| --- | --- | --- |
| JBI Cohort Checklist | Non-randomized comparative cohort studies | 40 |
| JBI Case Series Checklist | Single-arm case series / feasibility studies / registries | 47 |
| JBI Case Report Checklist | Single-patient case reports | 3 |
| RoB 2 (Cochrane revised) | Randomized controlled trials | 1 |
| JBI Analytical Cross-Sectional Checklist | Simulation / cross-sectional learning-curve study | 1 |

## Table 1. JBI Cohort Checklist - non-randomized comparative studies (n = 40)

*Abbreviations: Y = Yes; N = No; U = Unclear. C1 same source population; C2 exposure measured similarly in both groups; C3 exposure measurement valid and reliable; C4 confounders identified; C5 confounding strategies stated; C6 participants free of outcome at study start; C7 outcomes measured in a valid and reliable way; C8 follow-up time sufficient; C9 follow-up complete; C10 incomplete follow-up addressed; C11 statistical methods appropriate.*

| **Study** | **Platform** | **C1** | **C2** | **C3** | **C4** | **C5** | **C6** | **C7** | **C8** | **C9** | **C10** | **C11** | **Overall concern** |
| --- | --- | --- | --- | --- | --- | --- | --- | --- | --- | --- | --- | --- | --- |
| Ruvolo et al., 2024 | HUGO | Y | Y | Y | U | N | Y | Y | Y | U | Y | U | Moderate |
| Ianieri et al., 2024 | HUGO | Y | Y | Y | U | N | Y | Y | Y | U | Y | U | Moderate |
| Mastrovito et al., 2025 | HUGO | Y | Y | Y | Y | Y | Y | Y | U | U | N | U | Low |
| Law et al., 2025 | HUGO | Y | Y | Y | Y | Y | Y | Y | Y | U | Y | U | Moderate |
| Vargas Castillo et al., 2025 | HUGO | Y | Y | Y | Y | Y | Y | Y | U | U | N | Y | Moderate |
| Komatsu et al., 2026 | HUGO | Y | Y | Y | Y | N | Y | Y | Y | U | Y | Y | Moderate |
| Nagata et al., 2025 | HUGO; Hinotori | Y | Y | Y | Y | N | Y | Y | Y | U | Y | U | Moderate |
| Matsuura et al., 2024 | HUGO; Hinotori; Da Vinci SP | Y | Y | Y | Y | N | Y | Y | Y | Y | N | U | Moderate |
| Fioccola et al., 2026 | HUGO; Versius | N | Y | Y | U | N | Y | Y | Y | U | N | U | High |
| Togami et al., 2024 | Hinotori | Y | Y | Y | Y | N | Y | Y | Y | U | N | Y | Moderate |
| Ichino et al., 2024 | Hinotori | Y | Y | Y | U | N | Y | Y | Y | U | N | U | Moderate |
| Togami et al., 2025 (EC+SLN) | Hinotori | Y | Y | Y | U | N | Y | Y | U | U | N | U | Moderate |
| Togami et al., 2025 (SCP) | Hinotori | Y | Y | Y | U | N | Y | Y | U | U | N | Y | Moderate |
| Fukumoto et al., 2025 | Hinotori | Y | Y | Y | U | N | Y | Y | Y | U | N | U | Moderate |
| Pi et al., 2025 | KANGDUO | Y | Y | Y | Y | N | Y | Y | U | U | N | Y | Moderate |
| Liu et al., 2025 | KANGDUO | Y | Y | Y | Y | N | Y | Y | Y | U | Y | U | Moderate |
| Zhao et al., 2025 | SHURUI | Y | Y | Y | U | N | Y | Y | Y | U | N | U | Moderate |
| Coussons et al., 2021 | Senhance | Y | Y | Y | U | N | Y | Y | U | U | N | U | Moderate |
| Clark et al., 2023 | Senhance | Y | Y | Y | Y | Y | Y | Y | Y | U | N | Y | Low |
| Kim et al., 2022 | Da Vinci SP | Y | Y | Y | Y | Y | Y | Y | U | U | N | U | Low |
| Lee et al., 2023 | Da Vinci SP | Y | Y | Y | U | N | Y | Y | U | U | N | U | Moderate |
| Seon et al., 2023 (vs Xi) | Da Vinci SP | Y | Y | Y | Y | Y | Y | Y | Y | Y | Y | U | Low |
| Kim et al., 2023 (myomectomy I) | Da Vinci SP | Y | Y | Y | U | N | Y | Y | Y | U | N | Y | Moderate |
| Kim et al., 2023 (myomectomy II) | Da Vinci SP | Y | Y | Y | U | N | Y | Y | U | Y | Y | U | Moderate |
| Seon et al., 2023 (vs laparotomy) | Da Vinci SP | Y | Y | Y | Y | Y | Y | Y | Y | U | Y | U | Moderate |
| Oh et al., 2023 | Da Vinci SP | Y | Y | Y | Y | N | Y | Y | Y | U | Y | U | Moderate |
| Park et al., 2023 | Da Vinci SP | Y | Y | Y | U | N | Y | Y | U | U | N | U | Moderate |
| Lee et al., 2024 | Da Vinci SP | Y | Y | Y | U | N | Y | Y | U | U | N | U | Moderate |
| Matsuura et al., 2024 (SP) | Da Vinci SP | Y | Y | Y | Y | N | Y | Y | Y | Y | N | U | Moderate |
| Erdemoglu et al., 2025 | Da Vinci SP | Y | Y | Y | Y | Y | Y | Y | Y | U | Y | Y | Low |
| Lee et al., 2025 (PSM) | Da Vinci SP | Y | Y | Y | Y | Y | Y | Y | Y | Y | Y | U | Low |
| Higuchi et al., 2025 | Da Vinci SP | Y | Y | Y | Y | Y | Y | Y | Y | U | N | U | Moderate |
| Ohwaki et al., 2025 | Da Vinci SP | Y | Y | Y | U | N | Y | Y | Y | U | N | U | Moderate |
| Ferrigni et al., 2025 | Da Vinci SP | Y | Y | Y | Y | Y | Y | Y | Y | U | N | Y | Low |
| Miyamura et al., 2025 | Da Vinci SP | Y | Y | Y | U | N | Y | Y | U | U | N | U | Moderate |
| Vizza et al., 2025 (EJSO) | Da Vinci SP | Y | Y | Y | U | N | Y | Y | U | Y | N | Y | Moderate |
| Vizza et al., 2025 (case-control) | Da Vinci SP | Y | Y | Y | U | Y | Y | Y | U | U | N | U | Moderate |
| Cucinella et al., 2025 | Da Vinci SP | Y | Y | Y | U | N | Y | Y | Y | U | Y | U | Moderate |
| Vizza et al., 2025 (CUSUM) | Da Vinci SP | Y | Y | Y | Y | Y | Y | Y | U | U | N | U | Low |
| Kanno et al., 2025 | Da Vinci SP | Y | Y | Y | Y | Y | Y | Y | Y | U | Y | U | Low |

*Color key: Green = Low concern; Yellow = Moderate concern; Red = High concern.*

## Table 2. JBI Case Series Checklist - single-arm series / feasibility studies / registries (n = 47)

*Abbreviations: Y = Yes; N = No; U = Unclear. S1 inclusion criteria clearly defined; S2 condition measured consistently; S3 condition identified with valid methods; S4 consecutive patient inclusion; S5 complete patient inclusion; S6 demographic information reported; S7 clinical information reported clearly; S8 outcomes/follow-up reported; S9 site/clinic information reported; S10 statistical analysis appropriate.*

| **Study** | **Platform** | **S1** | **S2** | **S3** | **S4** | **S5** | **S6** | **S7** | **S8** | **S9** | **S10** | **Overall concern** |
| --- | --- | --- | --- | --- | --- | --- | --- | --- | --- | --- | --- | --- |
| Liu et al., 2025 | CARINA | Y | Y | Y | U | Y | U | Y | Y | Y | Y | Moderate |
| Gulz et al., 2025 | Dexter | Y | Y | Y | U | U | U | Y | Y | Y | Y | Low |
| Imboden et al., 2025 (prospective) | Dexter | Y | Y | Y | Y | Y | Y | Y | Y | Y | Y | Moderate |
| Imboden et al., 2025 (retrospective) | Dexter | Y | Y | U | U | U | U | Y | Y | Y | Y | Low |
| Gong et al., 2024 | EDGE SP1000 | Y | Y | U | U | Y | Y | Y | Y | U | Y | Moderate |
| Chen et al., 2024 | EDGE SP1000 | Y | Y | U | U | U | U | Y | Y | Y | Y | Moderate |
| Panico et al., 2023 (60 sacrocolpopexies) | HUGO | Y | Y | Y | U | U | Y | Y | Y | Y | U | Moderate |
| Panico et al., 2023 (CUSUM docking) | HUGO | Y | Y | Y | Y | U | Y | Y | Y | Y | U | Low |
| Monterossi et al., 2023 | HUGO | Y | Y | U | Y | U | U | Y | Y | U | Y | Moderate |
| Olsen et al., 2024 | HUGO | Y | U | U | Y | Y | Y | Y | Y | Y | Y | Moderate |
| Yagur et al., 2024 | HUGO | Y | Y | Y | U | U | Y | Y | Y | Y | Y | Moderate |
| Pavone et al., 2024 | HUGO | Y | Y | Y | Y | U | Y | Y | Y | Y | Y | Moderate |
| Afonina et al., 2024 | HUGO | Y | U | U | U | U | Y | Y | Y | Y | Y | Moderate |
| Yap et al., 2024 | HUGO | Y | U | U | Y | U | U | Y | Y | Y | U | Moderate |
| Gioe et al., 2024 | HUGO | Y | U | U | U | Y | U | Y | Y | Y | Y | Moderate |
| Sakamoto et al., 2025 | HUGO | Y | U | U | U | U | Y | Y | Y | U | Y | Moderate |
| Nozaki et al., 2025 | HUGO | Y | Y | Y | U | U | Y | Y | Y | Y | Y | Moderate |
| Anagani et al., 2025 | HUGO | Y | U | U | U | U | Y | Y | Y | Y | Y | Moderate |
| Albertus-Bofarull et al., 2026 | HUGO | Y | Y | Y | Y | Y | Y | Y | Y | Y | Y | Moderate |
| Togami et al., 2023 | Hinotori | Y | Y | Y | U | U | Y | Y | Y | Y | Y | Moderate |
| Lowenstein et al., 2020 | Hominis | Y | Y | Y | U | U | Y | Y | Y | Y | Y | Moderate |
| Lowenstein et al., 2021 | Hominis | Y | Y | Y | U | Y | Y | Y | Y | Y | Y | Moderate |
| Mercoli et al., 2026 | Maestro | Y | Y | Y | Y | U | Y | Y | Y | Y | Y | Low |
| Chang et al., 2024 | SHURUI | Y | Y | Y | Y | Y | Y | Y | Y | Y | U | Low |
| Hu et al., 2024 | SHURUI | Y | Y | Y | U | U | Y | Y | Y | Y | U | Low |
| Gueli Alletti et al., 2018 | Senhance | Y | Y | Y | U | U | Y | Y | Y | U | Y | Moderate |
| Samalavicius et al., 2020 | Senhance | Y | Y | Y | Y | U | Y | Y | Y | Y | U | Moderate |
| Sassani et al., 2022 | Senhance | Y | Y | Y | U | U | Y | Y | Y | Y | Y | Moderate |
| Abendstein et al., 2024 | Senhance | Y | U | U | U | U | Y | Y | Y | Y | Y | Moderate |
| Staib et al., 2025 | Senhance | Y | U | U | U | U | U | Y | U | U | U | Moderate |
| Abendstein et al., 2025 | Senhance | Y | U | Y | U | U | Y | Y | Y | Y | U | Moderate |
| Sighinolfi et al., 2025 | Toumai | Y | Y | Y | Y | Y | Y | Y | Y | Y | Y | Moderate |
| Puntambekar et al., 2021 | Versius | U | Y | Y | U | Y | Y | Y | Y | Y | Y | Moderate |
| Borse et al., 2022 | Versius | Y | Y | Y | U | U | Y | Y | U | Y | Y | Low |
| Soumpasis et al., 2023 | Versius | Y | Y | Y | U | U | Y | Y | Y | Y | U | Moderate |
| Sighinolfi et al., 2024 | Versius | Y | Y | Y | Y | Y | U | Y | Y | Y | U | Moderate |
| Panico et al., 2025 | Versius | Y | U | U | U | U | U | Y | Y | Y | U | Moderate |
| Sadlecki et al., 2025 | Versius | Y | Y | U | U | U | U | Y | Y | Y | U | Moderate |
| Uccella et al., 2025 | Versius | Y | Y | Y | Y | Y | Y | Y | Y | Y | Y | Moderate |
| Shin et al., 2020 | Da Vinci SP | Y | Y | U | U | U | Y | Y | Y | Y | Y | Moderate |
| Kwak et al., 2022 | Da Vinci SP | Y | Y | Y | Y | U | U | Y | Y | Y | Y | Moderate |
| Lee et al., 2022 | Da Vinci SP | Y | Y | Y | U | U | U | Y | Y | Y | Y | Moderate |
| Guan et al., 2024 | Da Vinci SP | Y | Y | Y | U | U | Y | Y | Y | Y | Y | Moderate |
| Ashmore et al., 2024 | Da Vinci SP | Y | Y | Y | U | U | Y | Y | Y | Y | Y | Moderate |
| Chiofalo et al., 2025 | Da Vinci SP | Y | Y | Y | U | U | Y | Y | Y | Y | Y | Moderate |
| Vizza et al., 2025 (R-SPH pilot) | Da Vinci SP | Y | Y | Y | U | U | Y | Y | U | U | Y | Moderate |
| Alwafai et al., 2025 | Da Vinci SP | Y | Y | U | U | U | Y | Y | Y | Y | Y | Moderate |

*Color key: Green = Low concern; Yellow = Moderate concern; Red = High concern.*

## Table 3. JBI Case Report Checklist - single-patient case reports (n = 3)

*Abbreviations: Y = Yes; N = No; U = Unclear. R1 patient demographics reported; R2 patient history and timeline presented; R3 current clinical condition at presentation described; R4 diagnostic tests or methods described; R5 intervention/treatment procedure described; R6 post-intervention condition described; R7 adverse or unanticipated events reported; R8 key takeaway lessons presented.*

| **Study** | **Platform** | **R1** | **R2** | **R3** | **R4** | **R5** | **R6** | **R7** | **R8** | **Overall concern** |
| --- | --- | --- | --- | --- | --- | --- | --- | --- | --- | --- |
| Alkatout et al., 2024 | Dexter | Y | U | Y | U | Y | Y | Y | Y | Moderate |
| Pazzaglia et al., 2025 | Toumai | Y | U | Y | U | Y | Y | Y | Y | Moderate |
| Pasquini et al., 2026 | Toumai | Y | Y | Y | Y | Y | Y | Y | Y | Moderate |

## Table 4. RoB 2 - randomized controlled trial (n = 1)

*RoB 2 domains: RND = bias arising from the randomization process; DEV = bias due to deviations from intended interventions; MISS = bias due to missing outcome data; MEAS = bias in outcome measurement; SEL = bias in selection of the reported result.*

| **Study** | **Platform** | **RND** | **DEV** | **MISS** | **MEAS** | **SEL** | **Overall judgment** |
| --- | --- | --- | --- | --- | --- | --- | --- |
| Li et al., 2025 | KANGDUO | Some concerns | Low risk | Low risk | Low risk | Some concerns | Some concerns |

*Note: "Some concerns" in the overall judgment reflects uncertainties in the randomization process (allocation concealment not fully described) and in the selection of reported results. The trial was conducted across multiple Chinese centers; the noninferiority margin and primary endpoint (lymph node yield) were pre-specified. No serious deviation concerns were identified.*

## Table 5. JBI Analytical Cross-Sectional Checklist - simulation/learning-curve study (n = 1)

*Abbreviations: Y = Yes; N = No; U = Unclear. X1 inclusion criteria clearly defined; X2 subjects and setting described in detail; X3 exposure measured validly and reliably; X4 standard objective criteria used for condition measurement; X5 confounding factors identified; X6 strategies to deal with confounding factors stated; X7 outcomes measured validly and reliably; X8 appropriate statistical analysis used.*

| **Study** | **Platform** | **X1** | **X2** | **X3** | **X4** | **X5** | **X6** | **X7** | **X8** | **Overall concern** |
| --- | --- | --- | --- | --- | --- | --- | --- | --- | --- | --- |
| Hutchins et al., 2019 | Senhance | Y | Y | Y | Y | Y | Y | Y | Y | Moderate |

*Note: The Hutchins et al. (2019) study used a simulation-based cross-sectional design (Fundamentals of Laparoscopic Surgery tasks) to assess early learning with the Senhance system. It was assessed with the JBI Analytical Cross-Sectional Checklist as the most appropriate available instrument. The moderate-concern rating reflects the absence of a control condition and the cross-sectional (non-longitudinal) measurement of learning, despite otherwise adequate methodological reporting.*

## Summary of risk-of-bias findings

Among the 92 included studies, risk of bias was assessed independently by two reviewers with disagreements resolved by consensus. One randomized trial was assessed with RoB 2, 40 non-randomized comparative studies with the JBI Cohort Checklist, 47 single-arm case series or feasibility studies with the JBI Case Series Checklist, three case reports with the JBI Case Report Checklist, and one simulation-based cross-sectional study with the JBI Analytical Cross-Sectional Checklist.

Among the 40 cohort studies, 9 were judged to have low concern, generally because they used formal propensity-score matching or other adjustment strategies and had acceptable outcome reporting; 30 were judged moderate concern, most commonly because of retrospective design, incomplete confounder adjustment, limited follow-up reporting, or unclear handling of missing data; and 1 was judged high concern because of inadequate comparability and underreported methodology. Among the 47 case series, 7 were judged to have low concern (prospective designs with adequate patient selection and outcome reporting), and 40 moderate concern (most commonly due to unclear consecutive enrollment, incomplete patient inclusion, or limited statistical reporting). All three case reports were judged to have moderate concern, reflecting the intrinsic design limitations of single-patient reporting regardless of reporting completeness. The single randomized trial (Li et al., 2025) raised some concerns under RoB 2, primarily in the domains of randomization process (allocation concealment not fully described) and selection of reported results. The single cross-sectional study (Hutchins et al., 2019) was judged to have moderate concern because of the absence of a control condition and the cross-sectional rather than longitudinal measurement of learning.

Overall, the available literature is largely composed of retrospective, single-center observational studies, most of which show low to moderate methodological quality. The main recurring limitations include the lack of concurrent control groups, insufficient adjustment for confounding factors, short and incompletely reported follow-up, and heterogeneity in outcome definition, measurement, and reporting. These limitations are reflected in the structured narrative synthesis and reinforce the need for cautious interpretation of all platform-specific findings, particularly for systems at early adoption stages where publication bias toward favorable outcomes is especially likely.
